# Analytical model of stress analysis for pipeline lowering-in during construction

**DOI:** 10.1371/journal.pone.0325123

**Published:** 2025-07-01

**Authors:** Xinfeng Pang, Lei Tong, Zhipeng Song, Xiao Sun, Zengcai Li, Lisong Zhang

**Affiliations:** 1 Engineering Technology Innovation Co., Ltd., PipeChina, Tianjin, China; 2 College of Pipeline and Civil Engineering, China University of Petroleum, Qingdao, China; China Construction Fourth Engineering Division Corp. Ltd, CHINA

## Abstract

During buried pipelines, two construction modes are used, namely, sinking and lowering pipeline into being-dug trench by self-weight, lifting and lowering pipeline into pre-dug trench by hoist. For pipeline sinking and lowering-in, the analytical model was derived especially considering the soil displacement at the end boundary of being-dug trench. For pipeline lifting and lowering-in, the control condition to calculate the lifting force was firstly given based on the extreme displacement of the pipeline. Then, finite difference on the pipeline deflection at each lifting point was performed to obtain the bending moment of the pipeline, and then the lifting point force was derived. Furthermore, the analytical model was established for lifting and lowering-in. By the finite element model and on-site experiment, the analytical models were validated. Results indicated that: (1) taking the length of arched segment, the length of suspended segment, the maximum stress and the bending moment as comparison variables, the maximum errors were 5.56%, 5.96%, 5.35%, 7.36% between the sinking and lowering-in model and the finite element model, while were 8.79%, 4.27%, 8.68%, 8.72% between the sinking and lowering-in model and the on-site experiment; (2) the maximum errors between the lifting and lowering model and finite element model were 7.63%, 8.59%, 3.74%, 6.44%, 9.51% and 8.13%, considering the lifting force and pipeline stress in the vertical plane, the lifting force and pipeline stress in the horizontal plane, and the combined lifting force and combined stress as comparison parameters, and meanwhile the analytical results showed the overall agreement to numerical model at the trench-touched point and the ground-departed point, with the relative errors of 8.59% and 3.68% (in the vertical plane), 5.73% and 4.39% (in the horizontal plane), 6.85% and 4.12% (combined stress), respectively.

## 1 Introduction

Due to the rapid construction of a cross-country pipeline [1,2], pipeline lowering into the trench (i.e., pipeline lowering-in) as a construction technology is being more and more important. Pipeline lowering-in can be primarily divided into two modes, namely, sinking and lowering-in by self-weight, lifting and lowering-in by hoist. Through sinking and lowering pipeline into the being-dug trench bottom by pipeline self-weigh [3–6], this approach provides a series of advantages, including low organizational complexity, minimal temporary land occupation, simple construction operation and significant economic benefit, making it particularly suitable in areas with poor foundation bearing capacity, such as water networks, deserts and swamps. Different to sinking and lowering-in, lifting and lowering-in is characterized by the quick operation speed, streamlined workflow and technical maturity, which makes it being the most widely-used approach during pipeline lowering-in. These two approaches show a good complementary, having been extensively applied in China-Russia East Natural Gas Pipeline project. However, these two approaches induce substantial bending displacement during the lowering-in, leading to localized tensile/ compressive strains and resulting in the strength failure (or even fracture) or buckling wrinkle [7], posing safety risks to pipeline integrity. Consequently, performing stress analysis during pipeline lowering-in would hold significant benefits for addressing the complex deformation challenges in large-diameter pipeline construction.

Extensive researches have been conducted on stress analysis for sinking and lowering-in by pipeline self-weight. Lan et al. [8] developed numerical model for sinking and lowering-in using surface-to-surface contact method, obtaining pipeline stress distributions under different conditions. Shi et al. [9] investigated the effects of soil properties, pipe wall thickness, support pier locations, presence/ absence of piers, and trench depth on pipeline suspended length and maximum stress by finite element model during sinking and lowering-in. Meng et al. [10] established stress analysis models for straight and curved pipeline segments during sinking and lowering-in using ANSYS with soil springs to simulate pipe-soil interaction, determining critical trench depths. Wang et al. [11] simplified the mechanical model of sinking and lowering-in as a statically indeterminate beam, derived expressions for the maximum stress under different trench depths, and validated results through finite element model. Gu et al. [12] conducted finite element simulations to discuss the feasibility of sinking and lowering-in. Liu et al. [13,14] analyzed stress distributions during sinking and lowering-in for the China-Russia East Pipeline, evaluated pipeline safety by stress strength criteria, and proposed feasibility assessment standards.

In addition, some scholars have achieved notable progress in stress analysis for pipeline lifting and lowering-in. Duan et al. [15] derived pipeline stress equation using beam model, demonstrating that pipeline stress can be effectively controlled by adjusting one or two lifting forces. Hwang et al. [16] developed segmented pipeline models using elastic foundation beam and Euler-Bernoulli beam to conduct pipeline stress analysis. Wang et al. [17] proposed closed-form solutions for bending stresses in vertical and horizontal planes during lifting and lowering-in. Sen et al. [18] simulated pipeline lowering-in for Trans Canada in Ontario using PIPLIN software, while Carneiro et al. [19] found that optimized lifting schemes reduced pipeline stress when decreasing lifting point spacing. Zhang et al. [20] established analytical model for horizontal bending and finite element model for vertical bending, along with calculation method of pipeline combined stress. Liu et al. [21] identified key control parameters (number of lifting points, relative heights between lifting points, trench depth) and correlated number of lifting points with pipe diameter and wall thickness. Scott et al. [22] developed stress analysis method under combined bending-torsion loads. Alexander and Scrivner [23] calculate stresses in girth welds through the finite element model during lifting and lowering-in construction, especially considering a series of parameters such as the lift height, the stress concentration factor, the residual stress. Additionally, researches on submarine pipeline S-lay and J-lay methods [24–27] provided valuable guidelines for lifting and lowering-in modeling.

Despite the above advancements, there are still limitations to be mentioned. Firstly, for sinking and lowering-in, finite element models were excessively used, resulting in the analytical model not well being developed. Although finite element model can provide better calculation accuracy, it consumes larger simulation resource, which means that the analytical model has sufficient necessity to be developed. Secondly, for lifting and lowering-in, calculation of lifting force belongs to a multi-solution problem, but in current researches no control condition was proposed to solve the lifting force. Meanwhile, the stress model in the vertical plane was given but the one in the horizontal plane was seldom established, therefore difficult to obtain the combined stress for the lifting and lowering-in.

To address the above gaps, the analytical stress model was developed for sinking and lowering-in by integrating deflection curve equation and deformation compatibility condition, especially considering the soil displacement at the end boundary of being-dug trench due to pipeline self-weight. Meanwhile, the other analytical stress model was established for lifting and lowering-in by enforcing control condition with zero-moment constraints at first and last lifting points. Finally, analytical models were validated by the finite element model and on-site experiment.

## 2 Stress analysis for sinking and lowering-in

The pipeline sinking and lowering-in is a construction technique that utilizes the pipeline self-weight to lowering pipeline into the being-dug trench. To achieve sinking and lowering-in, the pipeline was transported to the construction site using floating equipment, and then sinking and lowering pipeline into the trench bottom by pipeline self-weight to complete pipeline installation. During trench excavation, two excavators are initially deployed symmetrically on both sides of the pipeline to excavate the trench. Once the trench reaches a certain length, the pipeline begins to sink under self-weight. Continued excavation allows the pipeline to touch to the trench bottom, thereby completing the entire pipeline sinking and lowering-in process.

In mechanics, sinking and lowering-in inherently renders it particularly suitable for large-diameter pipelines with substantial self-weight. Compared to the additional complexity caused by the heavy self-weight of large-diameter pipelines during lifting and lowering-in, the sinking and lowering-in exhibits special advantages for pipeline construction.

### 2.1 Physical model

**Fig 1:**
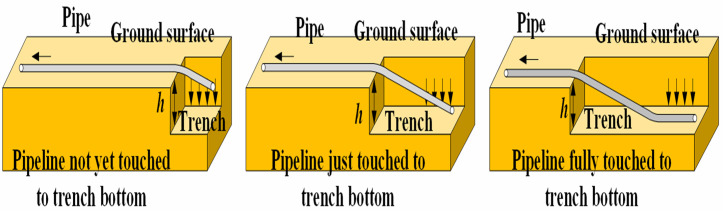
Physical model.

The sinking and lowering-in was illustrated in Fig 1, and can be divided into bilateral and unilateral excavations [28]. Given that bilateral excavation is predominantly employed in engineering practices, in this analysis bilateral excavation was defaulted as the basic configuration to be analyzed.

**Fig 2:**
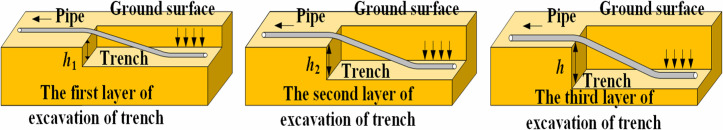
Layered excavation of trench.

Note that, as stipulated in Q/SY GDJ 0387–2014 [28], layered excavation of trench is recommended when the maximum stress in the steel pipe exceeds 80% of the minimum yield strength (SMYS), as shown in Fig 2.

### 2.2 Mechanical model

**Fig 3:**
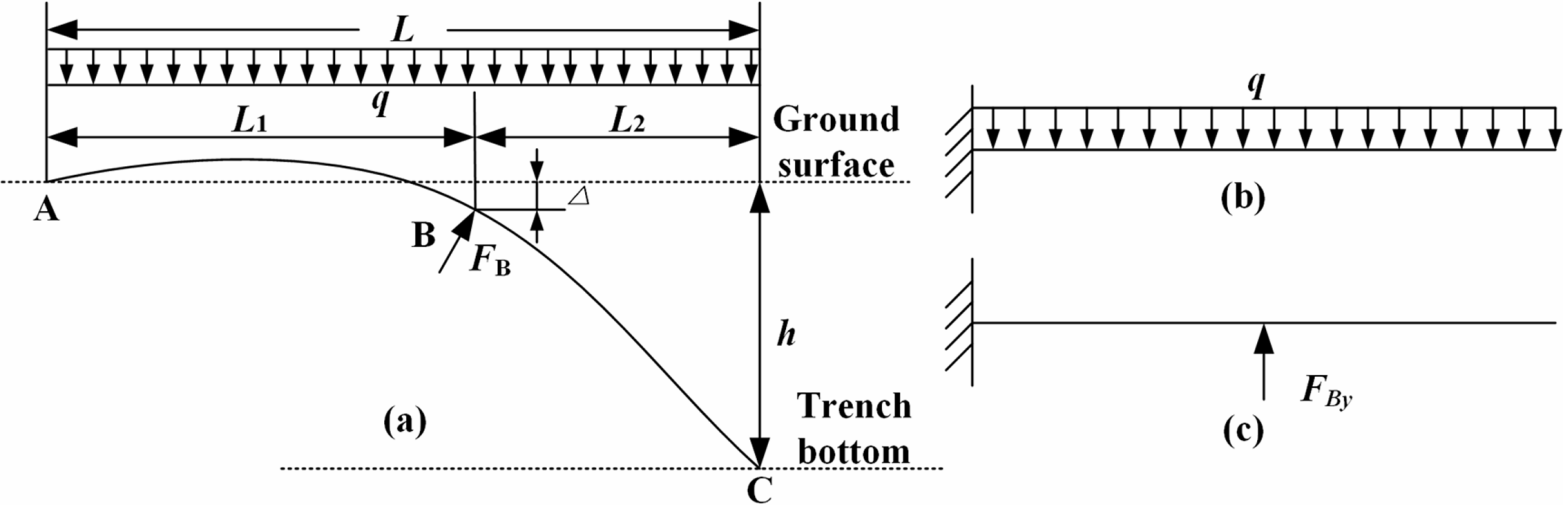
Stress analysis model during sinking and lowering-in.

The constraint at the trench excavation end (Point B) was treated as a contact surface constraint, resulting in *F*_B_ perpendicular to the pipeline axis at Point B. Notably, due to the pipeline self-weight, the soil at the excavation end undergoes compression during lowering-in, hereby generating compression displacemen Δ, which was considered as a critical distinction from prior studies [8–14,28]. In Fig 3, *L*_1_ is the length of the displacement segment on the ground (i.e., the length of the arched segment), m; *L*_2_ is the length of the suspended segment, m.

Based on the force-displacement relationship of the cantilever beam in Fig 3 and the deformation compatibility condition $\omega_{B}=\Delta$, the following expression was established:


$$\frac{qL_{1}^{2}}{24EI}\left(L_{1}^{2}-4LL_{1}+6L^{2}\right)-\frac{F_{By}L_{1}^{3}}{3EI}-\frac{F_{Bx}\Delta L_{1}^{2}}{2EI}=\Delta$$ (1)

where *q* is the self-weight per unit length of the pipe, N/m; *E* is the modulus of elasticity of the pipe, Pa; *I* is the moment of inertia of the pipe cross-section, m^4^; *ω*_B_ is the deflection at the excavation end B of trench, m; Δ is the compressive displacement of the soil at the excavation end B (i.e., the deflection of the pipeline at point B), m; *F*_*By*_ is the force component in the direction *y* provided by *F*_B_, N; *F*_*Bx*_ is the force component of the direction *x* provided by *F*_B_, N (during sinking and lowering-in, there is friction force induced by the pipeline to the ground, and the friction force is combined to *F*_*Bx*_ to constitute a force couple that inhibits the pipeline sinking and lowering-in, equaling to *F*_*Bx*_Δ); *L* is the total length of the deformed segment, m.

Integrating Eq. (1) to generate:


$$F_{By}=\frac{q}{8L_{1}}\left(L_{1}^{2}-4LL_{1}+6L^{2}\right)-\frac{3EI\Delta }{L_{1}^{3}}-\frac{3F_{Bx}\Delta }{2L_{1}}$$ (2)

Considering the equilibrium equation, Eq. (1) was re-written as Eq. (3) based on $\sum M_{A}\left(F\right)$=0 and *M*_A_ = 0.


$$\frac{1}{2}qL^{2}-F_{By}L_{1}-F_{Bx}\Delta =0$$ (3)

where *M*_A_ is the moment at the fixed end A, N·m; ∑*M*_A_(**F**) is the sum of the moments of the whole system when point A is the center of moment, N·m.

Substituting Eq. (2) into Eq. (3) yields:


$$\frac{1}{2}qL^{2}+\frac{3EI\Delta }{L_{1}^{2}}-\frac{q}{8}\left(L_{1}^{2}-4LL_{1}+6L^{2}\right)+\frac{1}{2}F_{Bx}\Delta =0$$ (4)

From the force-displacement relationship of the cantilever beam [29–32] and the deformation coordination condition, the pipeline deflection *ω*_C_ when the pipeline just touched to the trench bottom is equal to the trench depth *h*, i.e., *ω*_C_ = *h*, seeing Eq. (5):


$$\frac{qL^{4}}{8EI}-\frac{F_{By}L_{1}^{2}}{6EI}\left(3L-L_{1}\right)-\frac{F_{Bx}L_{1}\Delta }{EI}\left(L-\frac{L_{1}}{2}\right)=h$$ (5)

where *h* is the trench depth, m; *ω*_C_ is the pipeline deflection when pipeline just touched to the trench bottom, m; $F_{Bx}$ can be calculated by Eq. (6):


$$F_{Bx}=F_{By}\tan\theta$$

$$\theta =\frac{qL_{1}^{3}}{6EI}-\frac{qLL_{1}^{2}}{2EI}+\frac{qL^{2}L_{1}}{2EI}-\frac{F_{By}L_{1}^{2}}{2EI}-\frac{F_{Bx}\Delta L_{1}}{EI}$$ (6)

In particular, Δ in Eq. (1) can be expressed as the function of $F_{By}$ by the soil-spring model, as shown in Eq. (7):


$$\Delta =\frac{F_{By}}{\gamma hN_{q}D+0.5\gamma hN_{\gamma }D^{2}}$$ (7)

where γ is the weight of the soil, kN/m^3^; D is the diameter of the pipe, m; $N_{q}$, $N_{\gamma }$ is the compressive capacity factor of soil, related to the friction angle of soil [33].

Using Eqs. (1), (3), (5), (6) and (7), the variables of *L*, *L*_1_, Δ, $F_{By}$ and $F_{Bx}$ can be solved. After obtaining these parameters, the maximum bending moment of the pipeline during sinking and lowering-in can be calculated, occurring at the excavation end B, seeing Eq. (8):


$$M_{\max}=\frac{1}{2}qL_{2}^{2}$$ (8)

Further, the maximum bending stress of the pipeline during the sinking and lowering-in can be expressed as Eq. (9):


$$\sigma_{\max}=\frac{M_{\max}D}{I}$$ (9)

where $M_{\max}$is the maximum bending moment of the pipeline, N·m; $\sigma_{\max}$ is the maximum bending stress of the pipeline, Pa.

## 3 Stress analysis for lifting and lowering-in

### 3.1 Stress from bending in the vertical plane

#### 3.1.1 Extreme scenario of pipeline displacement.

**Fig 4:**
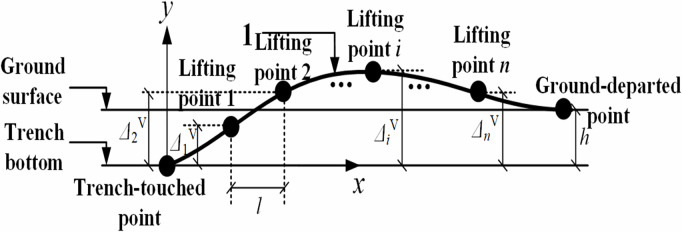
Pipeline displacement in the vertical plane.

During vertical lifting operations, the pipeline undergoes flexural deformation under the applied forces at each lifting point, as illustrated in Fig 4 below.

**Fig 5:**
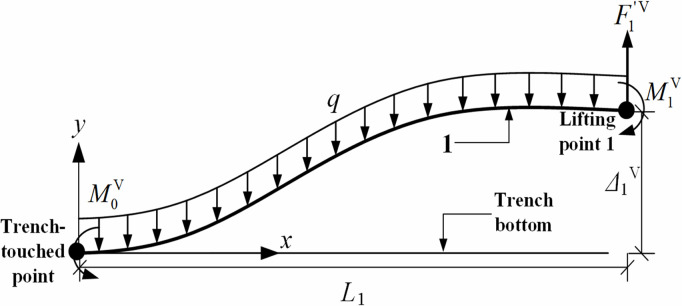
Mechanics model of pipeline segment between the trench-touched point and the first lifting point.

Based on Fig 4, the mechanical model was established for the pipeline segment between the trench-touched point and the first lifting point, and the internal forces acting on this pipeline segment were calculated, as shown in Fig 5, where q represents the pipeline self-weight per unit length; $F_{1}^{\prime \text{V}}$ represents the sectional shear force; $M_{1}^{V}$ represents the sectional bending moment.

**Fig 6:**
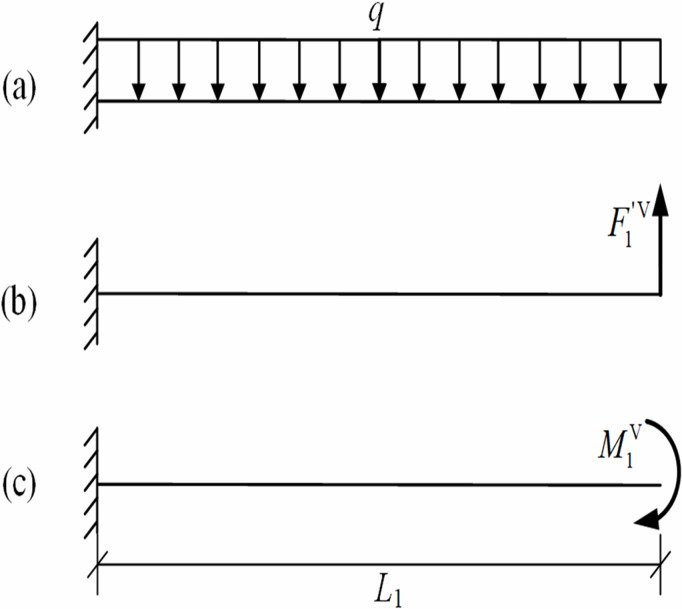
Simplified model forFig 7.

Considering the restraining effect of the remote pipeline on the trench-touched point, the trench-touched point was assumed to be fixed-end constraint. Under this condition, the mechanical model in Fig 5 was simplified as the one in Fig 6 based on the linear superposition principle.

The deflection curve equation for the pipeline segment between the trench-touched point and the first lifting point was obtained using the linear superposition principle, as shown in Eq. (10):


$${\omega_{x}}^{V}=\frac{F_{1}^{\prime \text{V}}x^{2}}{6EI}\left(3L_{1}-x\right)-\frac{qx^{2}}{24EI}\left(x^{2}-4L_{1}x+6{L_{1}}^{2}\right)-\frac{M_{1}^{V}x^{2}}{2EI},0\leq x\leq L_{1}$$ (10)

where ${\omega_{x}}^{V}$ is the deflection of the pipeline between the trench-touched point and the first lifting point, m; $F_{1}^{\prime \text{V}}$ is the shear force of the pipeline cross-section at the first lifting point, N; $L_{1}$ is the distance between the trench-touched point and the first lifting point, m; $M_{1}^{V}$ is the cross-sectional bending moment at the first lifting point, N·m; *q* is the self-weight of the pipe per unit length, equaling to $\rho g\frac{\pi \left(D^{2}-d^{2}\right)}{4}$, N/m; Dis the outer diameter of the pipe, m; dis the inner diameter of the pipe, m; *E* is the modulus of elasticity of the pipe, Pa; *I* is the moment of inertia of the pipe cross-section, equaling to $\frac{\pi \left(D^{4}-d^{4}\right)}{64}$, m^4^; x = 0 is the position at the trench-touched point, and *x*-direction is the direction of the pipeline axis; $x=L_{1}$ is the position of the first lifting point.

Solving the first-order derivative of the deflection equation to calculate the rotation angle ${\theta_{1}}^{V}$ of pipeline cross-section at the first lifting point, as seen in Eq. (11):


$${\theta_{1}}^{V}=\frac{F_{1}^{\prime \text{V}}{L_{1}}^{2}}{2EI}-\frac{q{L_{1}}^{3}}{6EI}-\frac{M_{1}^{V}L_{1}}{EI}$$ (11)

where ${\theta_{1}}^{V}$ is the rotation angle of pipeline cross-section at the first lifting point, rad;

Assuming that the shear force at the first lifting point equals the self-weight of the pipe segment, i.e., $F_{1}^{\prime \text{V}}=qL_{1}$, the pipeline displacement at the first lifting point (i.e., the free end of pipeline in Fig 6) was considered as two extreme scenarios (the actual pipeline displacement is between these two extreme scenarios) [15].

The first extreme scenario is that the rotation angle of pipeline cross-section at the first lifting point is 0, i.e., ${\theta_{1}}^{V}=0$. Using this condition, $M_{1}^{V}=\frac{qL_{1}^{2}}{3}$ and ${M_{0}}^{V}=\frac{q{L_{1}}^{2}}{6}$, where ${M_{0}}^{V}$ is the pipeline bending moment at the trench-touched point, N·m;

The second extreme scenario is that the bending moment of pipeline cross-section at the first lifting point is 0, i.e., $M_{1}^{V}=0$. Using this condition, ${M_{0}}^{V}=\frac{q{L_{1}}^{2}}{2}$.

Comparing the first and the second extreme scenarios, it can be seen that the bending moment at the position of the trench-touched point in the second extreme scenario is larger than the ones at the trench-touched point and the first lifting point in the first extreme scenario, which means the greater bending stress of the pipeline for the second extreme scenario. Consequently, the subsequent analysis would be based on the second extreme scenario.

In the next section 3.1.2, the acting forces of the first and last lifting points would be emphatically discussed under the second extreme scenario, to determine the control condition of calculating the lifting point force.

#### 3.1.2 Control condition of vertical lifting force.

**Fig 7:**
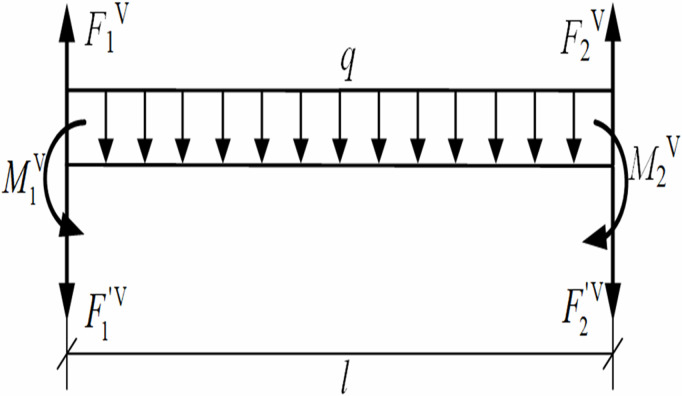
Mechanics model of pipeline segment between the first and second lifting point in the vertical plane.


Based on Fig 4, the mechanical model was established for the pipeline segment between the first and second lifting points, as shown in Fig 7, and the equilibrium equation $\sum {M_{2}}^{V}\left(F\right)=0$ for the pipeline segment between the first and second lifting points was derived as Eq. (12), where $\sum {M_{2}}^{V}\left(F\right)$ is the sum of the moments of the whole system when the second lifting point is the center of moment, N·m.

$${F_{1}}^{V}=\frac{M_{1}^{V}}{l}+F_{1}^{\prime \text{V}}-\frac{{M_{2}}^{V}}{l}+\frac{1}{2}ql$$ (12)

where ${F_{1}}^{V}$ is the lifting point force required in the vertical plane, N; *l* is the distance between two adjacent lifting points, m; ${M_{2}}^{V}$ is the bending moment of the pipeline cross-section at the second lifting point, N·m.

During the actual vertical lifting, the pipeline displacement at each lifting point was typically manifested as a convex upward and concave downward configuration, corresponding to the direction ($M_{1}^{V}$) illustrated in Fig 7. According to Eq. (12), when the cross-section moment $M_{1}^{V}$ at the first lifting point equals zero, the vertical lifting force ${F_{1}}^{V}$ at this location was minimized. This revealed that imposing the condition of zero moment at the first lifting point led to an increased bending moment at the pipeline trench-touched point, while simultaneously achieving the minimum acting force required at the first lifting point.

It is noteworthy that in Eqs. (10)–(11), the distance $L_{1}$ between the trench-touched point and the first lifting point remains an unknown variable. Therefore, it is necessary to calculate $L_{1}$ by incorporating deformation compatibility condition. By substituting $M_{1}^{V}=0$ and $x=L_{1}$ under the second extreme scenario into Eq. (10) to compute the deflection ${\omega_{1}}^{V}$ at the first lifting point, and then establishing deformation compatibility condition through ${\omega_{1}}^{V}={\Delta_{1}}^{V}$, the length $L_{1}$ of the pipeline segment was obtained as shown in Eq. (13):


L1=24EIΔ1V5q4\hfill\hfill\hfill (13)

where ${\Delta_{1}}^{V}$ is the vertical deflection of the first lifting point, i.e., the vertical distance from the first lifting point to the bottom of the trench, m.

**Fig 8:**
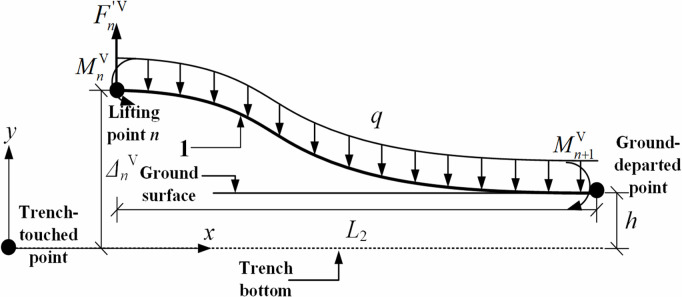
Mechanics model of pipeline between the last lifting point and the ground-departed point in the vertical plane.

By similar way, the mechanical model between the *n*th lifting point (i.e., the last lifting point) and the ground-departed point was established, as shown in Fig 8. Specifically, the mechanical model was also solved based on the condition that the bending moment at the *n*th lifting point is zero, i.e., $M_{n}^{V}=0$.

Using $\sum {M_{n}}^{V}\left(F\right)=0$ and ${M_{n}}^{V}=0$, it can be derived for Eqs. (14)–(15):


$$F_{n}^{\prime \text{V}}=qL_{2}$$ (14)

$${M_{n+1}}^{V}=\frac{q{L_{2}}^{2}}{2}$$ (15)

where $\sum {M_{n}}^{V}\left(F\right)$ is the sum of the moments of the whole system when the *n*th lifting point is the center of moment, N·m; ${M_{n}}^{V}$ is the bending moment at the *n*th lifting point, N·m; $F_{n}^{\prime \text{V}}$ is the pipe shear force at the *n*th lifting point in the vertical plane, N; ${M_{n+1}}^{V}$ is the pipe bending moment at the ground-departed point in the vertical plane, N·m; $L_{2}$ is the distance between the *n*th lifting point and the ground-departed point in the vertical plane, m; ${\Delta_{n}}^{V}$ is the vertical deflection of the *n*th lifting point, i.e., the vertical distance from the *n*th lifting point to the trench bottom, m; h is the trench depth, m.

From the deformation compatibility condition at the *n*th lifting point ${\omega_{n}}^{V}={\Delta_{n}}^{V}-h$ (where ${\omega_{n}}^{V}$ is the pipeline deflection at the *n*th lifting point), the length of the pipe segment between the *n*th lifting point and the ground-departed point can be derived, as presented in Eq. (16).


$$L_{2}=\sqrt[{4}]{\frac{24EI\left({\Delta_{n}}^{V}-h\right)}{5q}}$$ (16)

Similar to Eq. (12), the condition that the bending moment at the *n*th lifting point equals zero resulted in an increased bending moment at the ground-departed point, while simultaneously minimizing the vertical lifting force required at the *n*th lifting point.

Through comprehensive analysis, the boundary conditions requiring zero bending moments at the first and *n*th lifting points (i.e., the first and last lifting points) can be regarded as control conditions for calculation of lifting force and pipeline stress in the vertical plane.

#### 3.1.3. Calculation of vertical lifting force.

Referring to the differential equation of the deflection curve, the bending moment ${M_{i}}^{V}$ at the *i*th lifting point (where 2 ≤ *i *≤ *n*-1) was obtained by vertical deflections at each lifting point, as shown in Eq. (17):


$${M_{i}}^{V}=\frac{{\Delta_{i+1}}^{V}-2{\Delta_{i}}^{V}+{\Delta_{i-1}}^{V}}{l^{2}}EI$$ (17)

where ${M_{i}}^{V}$ is the bending moment at the *i*th lifting point in the vertical plane, N·m; ${\Delta_{i}}^{V}$ is the vertical deflection at the *i*th lifting point, i.e., the vertical distance from the *i*th lifting point to the bottom of the trench, m.

**Fig 9:**
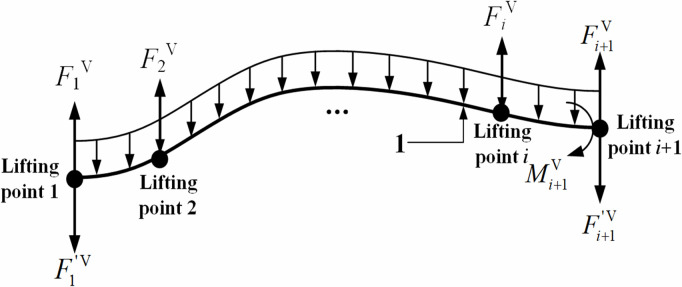
Mechanics model of pipeline segment between the first lifting point and the (*i* + 1)th lifting point in the vertical plane.


In detail, the vertical lifting force ${F_{1}}^{V}$ required at the 1st lifting point was derived based on the equilibrium equation $\sum {M_{1}}^{V}\left(F\right)=0$ of the pipe segment between the 1st and 2nd lifting points (seeing Fig 9), especially considering ${M_{1}}^{V}=0$, as shown in Eq. (18):

$${F_{1}}^{V}=F_{1}^{\prime \text{V}}-\frac{{M_{2}}^{V}}{l}+\frac{1}{2}ql$$ (18)

**Fig 10:**
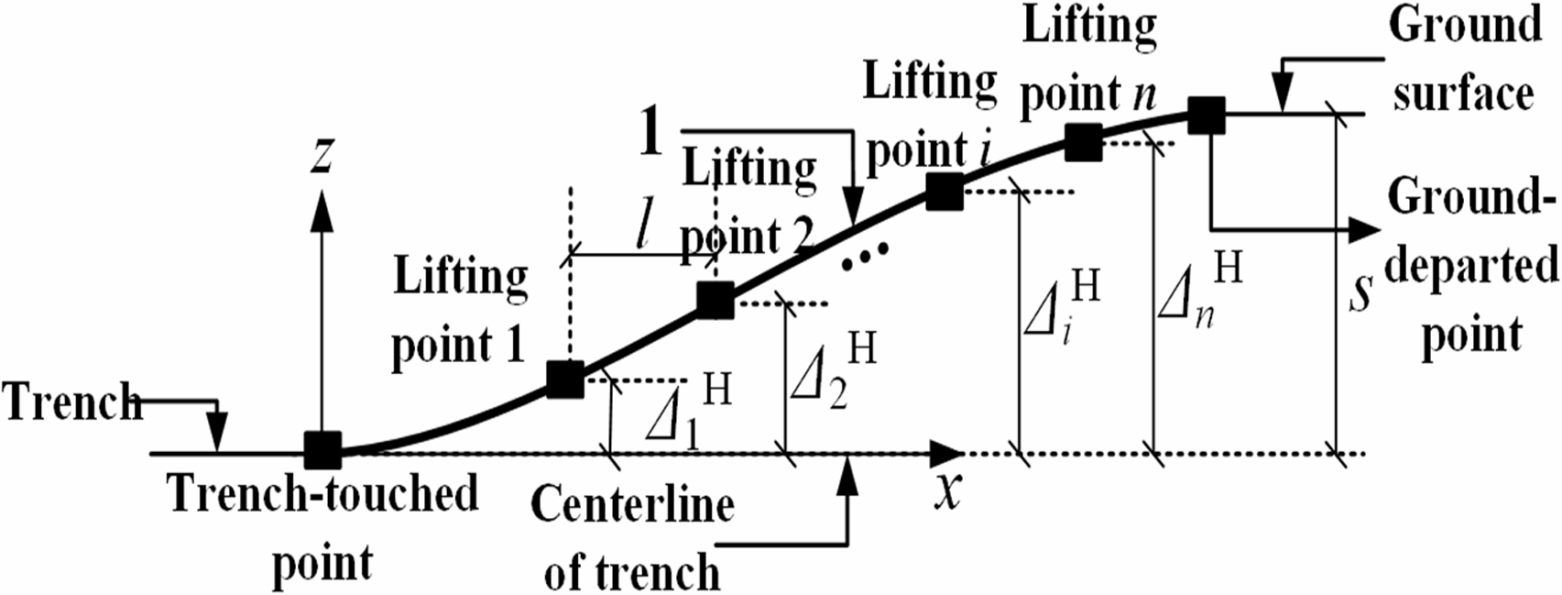
Pipeline displacement in the horizontal plane.


Similarly, considering the bending moment at the *n*th lifting point ${M_{n}}^{V}=0$, the vertical lifting force ${F_{n}}^{V}$ required at the *n*th lifting point was obtained based on the equilibrium equation $\sum {M_{n}}^{V}\left(F\right)=0$ of the pipe segment between the (*n*-1)th and *n*th lifting points (seeing Fig 10), as shown in Eq. (19):

$${F_{n}}^{V}=F_{n}^{\prime \text{V}}-\frac{{M_{n-1}}^{V}}{l}+\frac{1}{2}ql$$ (19)

where ${F_{n}}^{V}$ is the vertical lifting force required at the *n*th lifting point in the vertical plane, N; ${M_{n-1}}^{V}$ is the bending moment of the pipeline at the (*n*-1)th lifting point in the vertical plane, N·m.

Considering the pipe segment from the 1st lifting point to the (*i* + 1)th lifting point (seeing Fig 9), the vertical lifting force ${F_{i}}^{V}$ required at the *i*th lifting point was determined based on the equilibrium equation $\sum {M_{i+1}}^{V}\left(F\right)=0$, as shown in Eq. (20):


$${F_{i}}^{V}=\frac{\frac{1}{2}q{\left(il\right)}^{2}+F_{1}^{\prime \text{V}}il-\sum_{k=2}^{i}{F_{k-1}}^{V}(i-k+2)l-{M_{i+1}}^{V}}{l}\left(2\leq i\leq n-1\right)$$ (20)

where ${F_{i}}^{V}$ is the vertical lifting force required at the *i*th lifting point in the vertical plane, N; ${M_{i+1}}^{V}$ is the pipeline bending moment at the (*i* + 1)th lifting point in the vertical plane, N·m; ${F_{k-1}}^{V}$ is the vertical lifting force required at the (*k* − 1)th lifting point in the vertical plane, N.

#### 3.1.4 Stress analysis in the vertical plane.

Based on Eq. (17) and ${M_{0}}^{V}=\frac{q{L_{1}}^{2}}{2}$, ${M_{n+1}}^{V}=\frac{q{L_{2}}^{2}}{2}$, the pipeline bending stresses at each lifting point, trench-touched point, and ground-departed point in the vertical plane were calculated, as shown in Eq. (21):


$$\left\{ \begin{array}{c} {\sigma_{i}}^{V}=\frac{{M_{i}}^{V}D}{2I}\\ {\sigma_{0}}^{V}=\frac{{M_{0}}^{V}D}{2I}\\ {\sigma_{n+1}}^{V}=\frac{{M_{n+1}}^{V}D}{2I} \end{array}\right.$$ (21)

where $\sigma_{i}^{V}$ is the pipeline stress at the *i*th lifting point (2 ≤ *i* ≤ *n*), Pa; $\sigma_{0}^{V}$ is the pipeline stress at the trench-touched point, Pa; $\sigma_{n+1}^{V}$ is the pipeline stress at the ground-departed point, Pa.

### 3.2 Stress from bending in the horizontal plane

#### 3.2.1 Control condition of horizontal offset force.

Under the horizontal offset operation during lowering-in, the pipeline undergoes lateral deflection deformation under the horizontal offset force at each lifting point, as shown in Fig 10.

**Fig 11:**
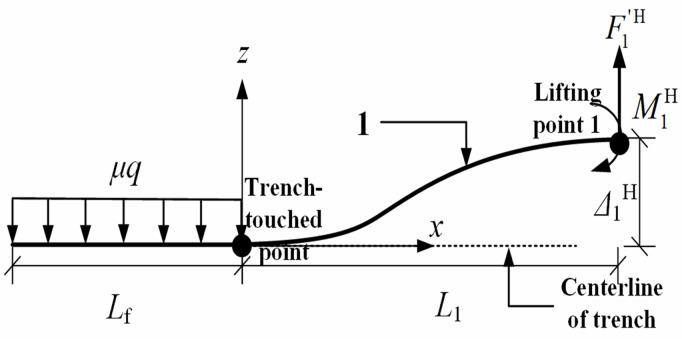
Mechanics model of pipeline between the trench-touched point and the first lifting point in the horizontal plane.

Due to the horizontal offset force at the first lifting point, the shear force $F_{1}^{\prime \text{H}}$ at the pipeline cross-section of the first lifting point is non-zero. Consequently, when truncating the pipeline at the first lifting point, a pipe-soil friction force is necessarily applied in the horizontal direction to maintain equilibrium with $F_{1}^{\prime \text{H}}$, as illustrated in Fig 11. Assuming $L_{f}$ to be the length of the pipeline subjected to static transverse friction within the trench in the horizontal plane, and then $F_{1}^{\prime \text{H}}=\mu qL_{f}$, where $F_{1}^{\prime \text{H}}$ denotes the shear force at the first lifting point in the horizontal plane, N; *μ* represents the pipe-soil friction coefficient in the horizontal plane, dimensionless.

Note that, it is necessary to meet the control condition for calculation of horizontal offset force, i.e., the bending moments at the 1st and *n*th lifting points are zero in the horizontal plane. Under this scenario, the bending moment ${M_{0}}^{H}$ at the trench-touched point was expressed as:


$${M_{0}}^{H}=F_{1}^{\prime \text{H}}L_{1}=\mu qL_{f}L_{1}$$ (22)

where ${M_{0}}^{H}$ is the pipeline bending moment at the trench-touched point in the horizontal plane, N·m.

**Fig 12:**
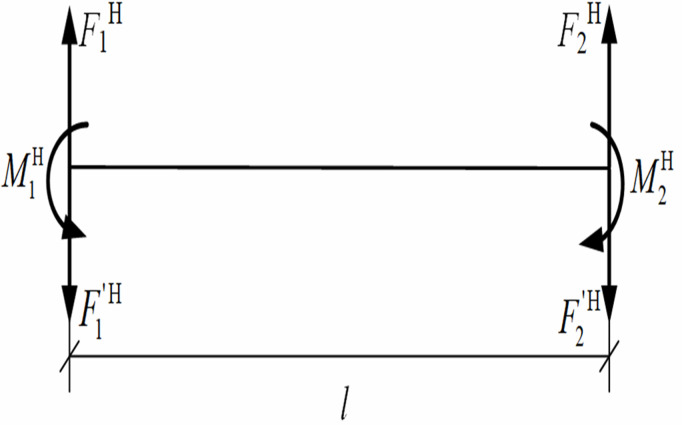
Mechanics model of pipeline between the first and second lifting points in the horizontal plane.


Especially, the horizontal offset force ${F_{1}}^{H}$ at the 1st lifting point was minimized when the sectional bending moment $M_{1}^{H}=0$ at the 1st lifting point, which was illustrated in Fig 12 and Eq. (23). Fig 12 showed the mechanical model between the 1st and 2nd lifting points in the horizontal plane, and Eq. (23) was derived based on $\sum {M_{2}}^{H}\left(F\right)=0$.

$${F_{1}}^{H}=\frac{{M_{1}}^{H}}{l}+F_{1}^{\prime \text{H}}-\frac{{M_{2}}^{H}}{l}$$ (23)

where $\sum {M_{2}}^{H}\left(F\right)$ is the sum of the moments of the whole system when the second lifting point is the center of moment in the horizontal plane, N·m; ${F_{1}}^{H}$ is the horizontal offset force required at the first lifting point in the horizontal plane, N; ${M_{2}}^{H}$ is the pipeline bending moment at the second lifting point in the horizontal plane, N·m.

During actual pipeline displacement in the horizontal plane, the pipeline displacement at each lifting point typically exhibits an forward convex and backward concave configuration, i.e., the direction shown by ${M_{1}}^{H}$ in Fig 12. Therefore, as derived from Eq. (23), when the sectional bending moment at the first lifting point satisfies $M_{1}^{H}=0$, the horizontal offset force ${F_{1}}^{H}$ at the first lifting point in the horizontal plane was minimized.

From Eq. (22), the length $L_{f}$ of the pipeline subjected to horizontal transverse static friction force within the trench in the horizontal plane remains unknown. Thus, $L_{f}$ is necessary to be calculated by incorporating deformation compatibility condition, as shown in Eq. (24):


$$\frac{F_{1}^{\prime \text{H}}{\left(L_{1}+L_{f}\right)}^{3}}{3EI}-\frac{\mu g{L_{f}}^{4}}{8EI}-\frac{\mu g{L_{f}}^{3}}{6EI}L_{1}={\Delta_{1}}^{H}$$ (24)

where ${\Delta_{1}}^{H}$ is the horizontal deflection at the first lifting point, i.e., the horizontal distance from the first lifting point to the trench centerline, m.

**Fig 13:**
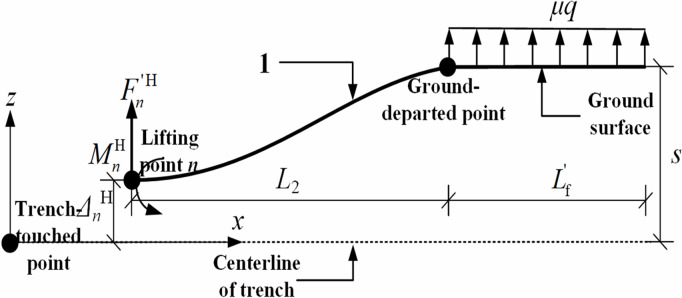
Mechanics model of pipeline between the last lifting point and the ground-departed point in the horizontal plane.


By similar way, the mechanical model between the *n*th lifting point (i.e., the last lifting point) and the ground-departed point was also be established in the horizontal plane, as illustrated in Fig 13. Notably, the mechanical model was solved based on the condition of zero bending moment at the *n*th lifting point in the horizontal plane, i.e., $M_{n}^{H}=0$. The parameter $L_{f}^{\prime }$ was derived from the deformation compatibility condition at the *n*th lifting point, as presented in Eq. (25).

$$\frac{F_{n}^{\prime \text{H}}{\left(L_{2}+{L_{\text{f}}}^{\prime }\right)}^{3}}{3EI}-\frac{\mu gL_{f}^{\prime \text{4}}}{8EI}-\frac{\mu gL_{f}^{\prime \text{3}}}{6EI}L_{2}=s-{\Delta_{n}}^{H}$$ (25)

Considering the following relation:


$$F_{n}^{\prime \text{H}}=\mu q{L_{\text{f}}}^{\prime }$$ (26)

The pipeline bending moment ${M_{n+1}}^{H}$ at the ground-departed point in the horizontal plane can be expressed as:


$${M_{n+1}}^{H}=F_{n}^{\prime \text{H}}L_{2}=\mu q{L_{\text{f}}}^{\prime }L_{2}$$ (27)

where $F_{n}^{\prime \text{H}}$ is pipeline shear force at the *n*th lifting point in the horizontal plane, N; ${L_{\text{f}}}^{\prime }$ is length of pipeline subjected to horizontal transverse static friction on ground surface in the horizontal plane, m; ${\Delta_{n}}^{H}$ is horizontal deflection at the *n*th lifting point location, i.e., horizontal distance from the *n*th lifting point to trench centerline, m; *s* is distance from pipeline axis to trench centerline at ground-departed point, m; s is distance from pipeline axis to trench centerline at ground-departed point, m; ${M_{n+1}}^{H}$ is pipeline bending moment at ground-departed point in the horizontal plane, N·m.

#### 3.2.2 Calculation of horizontal offset force.

Referencing to the differential equation of the deflection curve, the bending moment ${M_{i}}^{V}$ at the pipeline cross-section of the *i*th lifting point (2 ≤ *i* ≤ *n*-1) was obtained by horizontal deflections at each lifting point, as shown in Eq. (28):


$${M_{i}}^{H}=\frac{{\Delta^{H}}_{i+1}-2{\Delta^{H}}_{i}+{\Delta^{H}}_{i-1}}{l^{2}}EI$$ (28)

where ${M_{i}}^{H}$ is the bending moment at the *i*th lifting point in the horizontal plane, N·m; ${\Delta_{i}}^{H}$ is the horizontal deflection at the *i*th lifting point position (horizontal distance from the *i*th lifting point to trench centerline), m.

In addition, equilibrium equations for pipe segment between the 1st and 2nd lifting points and (*n*-1)th– *n*th lifting points in horizontal plane were given, namely, $\sum {M_{1}}^{H}\left(F\right)=0$, $\sum {M_{n}}^{H}\left(F\right)=0$. Combined with zero bending moment conditions at the 1st and *n*th lifting points ${M_{1}}^{H}=0$,${M_{n}}^{H}=0$), the horizontal offset forces ${F_{1}}^{H}$ and ${F_{n}}^{H}$ required at these positions were derived as Eqs. (29)–(30):


$${F_{1}}^{H}=\frac{{M_{2}}^{H}}{l}+F_{1}^{\prime \text{H}}$$ (29)

$${F_{n}}^{H}=\frac{{M_{n-1}}^{H}}{l}-F_{n}^{\prime \text{H}}$$ (30)

where ${F_{1}}^{H}$ is horizontal offset force required at 1st lifting point in horizontal plane, N; ${F_{n}}^{H}$ is horizontal offset force required at *n*th lifting point in horizontal plane, N; ${M_{2}}^{H}$ is bending moment at 2nd lifting point in horizontal plane, N·m; ${M_{n-1}}^{H}$ is bending moment at (*n*-1)th lifting point in horizontal plane, N·m.

**Fig 14:**
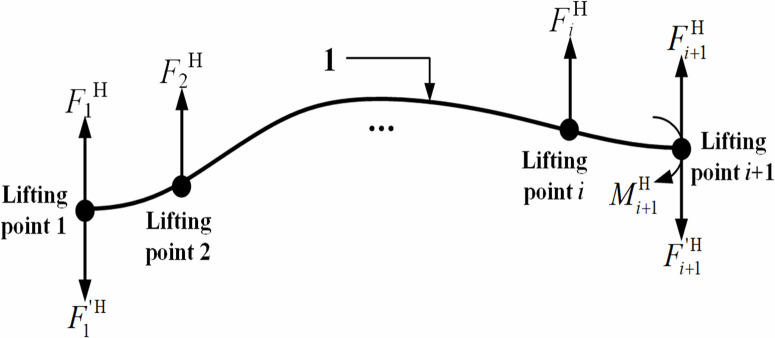
Mechanics model of pipeline between the first lifting point and the (*i* + 1)th lifting point in the horizontal plane.


Furthermore, the mechanical model was established for the pipe segment between the 1st and (*i* + 1)th lifting points, as shown in Fig 14. Through moment equilibrium equation about the (*i*+1)th lifting point $\sum {M_{i+1}}^{H}\left(F\right)=0$, the horizontal offset force ${F_{i}}^{H}$ required at the *i*th lifting point was obtained by Eq. (31):

$${F_{i}}^{H}=\frac{{M_{i+1}}^{H}+(F_{1}^{\prime \text{H}}-{F_{1}}^{H})il-\sum_{k=2}^{i}{F^{H}}_{k-1}(i-k+2)l}{l}\left(2\leq i\leq n-1\right)$$ (31)

where ${F_{i}}^{H}$ is horizontal offset force required at *i*${M_{i+1}}^{H}$ is pipeline bending moment at (*i*${F_{k-1}}^{H}$ is horizontal offset force required at (*k*-1)th lifting point in horizontal plane, N.

#### 3.2.3 Stress analysis in the horizontal plane.

From Eqs. (22), (27) and (28), the bending stresses at each lifting point, trench-touched point, and ground-departed point in the horizontal plane can be calculated as shown in Eq. (32):


$$\left\{ \begin{array}{c} {\sigma_{i}}^{H}=\frac{{M_{i}}^{H}D}{2I}\\ {\sigma_{0}}^{H}=\frac{{M_{0}}^{H}D}{2I}\\ {\sigma_{n+1}}^{H}=\frac{{M_{n+1}}^{H}D}{2I} \end{array}\right.$$ (32)

where $\sigma_{i}^{H}$ is the pipe stress at the *i*th (2 ≤ *i* ≤ n–1) lifting point in horizontal plane, Pa; $\sigma_{0}^{H}$ is the pipe stress at the trench-touched point in horizontal plane, Pa; $\sigma_{n+1}^{H}$ is the pipe stress at the ground-departed point in horizontal plane, Pa.

### 3.3 Combined lifting force and stress

After obtaining the vertical lifting force, the horizontal offset force and bending moments (including those at trench-touched and ground-departed points) in vertical and horizontal planes, the combined lifting forces and combined bending moments at each lifting point can be determined as Eqs. (33)–(34):


$$\left\{ \begin{array}{c} F_{i}^{T}=\sqrt{{\left({F_{i}}^{V}\right)}^{2}+{\left({F_{i}}^{H}\right)}^{2}}\\ F_{1}^{T}=\sqrt{{\left({F_{1}}^{V}\right)}^{2}+{\left({F_{1}}^{H}\right)}^{2}}\\ F_{n}^{T}=\sqrt{{\left({F_{n}}^{V}\right)}^{2}+{\left({F_{n}}^{H}\right)}^{2}} \end{array}\right.$$ (33)

$$\left\{ \begin{array}{c} M_{i}^{T}=\sqrt{{\left({M_{i}}^{V}\right)}^{2}+{\left({M_{i}}^{H}\right)}^{2}}\\ M_{1}^{T}=0\\ M_{n}^{T}=0\\ M_{0}^{T}=\sqrt{{\left({M_{0}}^{V}\right)}^{2}+{\left({M_{0}}^{H}\right)}^{2}}\\ M_{n+1}^{T}=\sqrt{{\left({M_{n+1}}^{V}\right)}^{2}+{\left({M_{n+1}}^{H}\right)}^{2}} \end{array}\right.$$ (34)

Furthermore, the pipe combined stress $\sigma_{i}^{T}$$\sigma_{1}^{T}$$\sigma_{n}^{T}$$\sigma_{0}^{T}$$\sigma_{n+1}^{T}$ at specified positions were calculated as Eq. (35):


$$\left\{ \begin{array}{c} \sigma_{i}^{T}=\frac{M_{i}^{T}D}{2I}\\ \sigma_{1}^{T}=0\\ \sigma_{n}^{T}=0\\ \sigma_{0}^{T}=\frac{M_{0}^{T}D}{2I}\\ \sigma_{n+1}^{T}=\frac{M_{n+1}^{T}D}{2I} \end{array}\right.$$ (35)

where $F_{i}^{T}$ is combined lifting force at the *i*th (2≤*i*≤*n*-1) lifting point, N; $F_{1}^{T}$ is combined lifting force at the 1st lifting point, N; $F_{n}^{T}$ is combined lifting force at the *n*th lifting point, N; $M_{i}^{T}$ is combined bending moment at the *i*th (2≤*i*≤*n*-1) lifting point, N·m; $M_{1}^{T}$ is combined bending moment at the 1st lifting point, N·m; $M_{n}^{T}$ is combined bending moment at the *n*th lifting point, N·m; $M_{0}^{T}$ is combined bending moment at the trench-touched point, N·m; $M_{n+1}^{T}$ is combined bending moment at the ground-departed point, N·m; $\sigma_{i}^{T}$ is pipe stress at the *i*th (2≤*i*≤*n*-1) lifting point, Pa; $\sigma_{1}^{T}$ is pipe stress at the 1st lifting point, Pa; $\sigma_{n}^{T}$ is pipe stress at the *n*th lifting point, Pa; $\sigma_{0}^{T}$ is pipe stress at the trench-touched point, Pa; $\sigma_{n+1}^{T}$ is pipe stress at the ground-departed point, Pa.

## 4 On-site engineering experiment

### 4.1 Experiment scheme

Experiment background: validating the analytical model for sinking and lowering-in by carrying out on-site engineering experiment that can collect the pipeline’s stress data and deflection data during the sinking and lowering-in.

Experiment location: Section I of the Hulin–Changchun Natural Gas Pipeline Project (Hulin starting station to Changchun compressor station), spanning from AA026-517m (horizontal continuous mileage 14 km + 800m) to AA026-117m (horizontal continuous mileage 15 km + 200m).

Monitoring technology and lowering-in mode of pipelines: the experiment length of pipelines of 200 m. Prior to the experiment, mileage markers were made every 10 meters along the pipeline, covering a range of 0 m to +200 m. The direction of sinking and lowering-in was preceded from 0 m to 200 m, with an excavation depth of 2.0 m.

**Fig 15:**
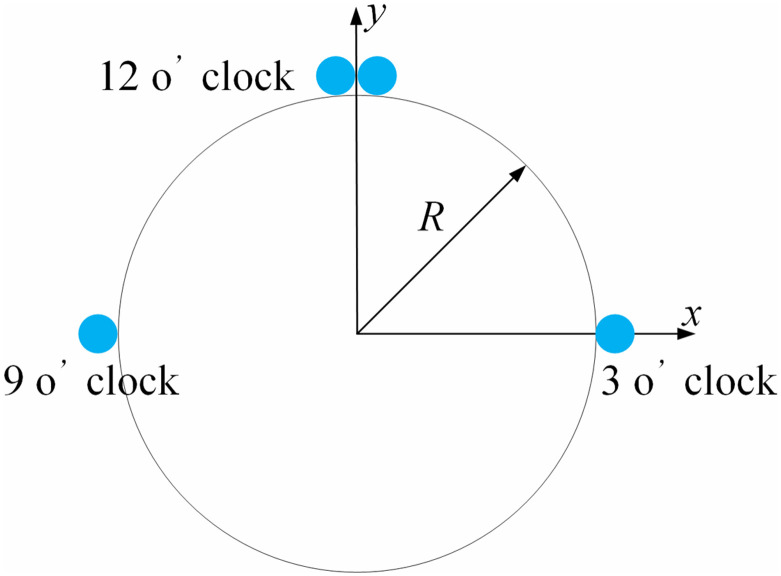
Stress monitoring scheme by distributed optical fiber.

Stress monitoring mode: distributed optical fibers adopted for monitoring pipeline stress are employed to monitor the distribution of axial stress along the pipeline. Distributed optical fibers were continuously installed along the axial direction of a 200-meter-long experimental pipeline. Specifically, one distributed optical fiber was deployed at each of the 3 o’clock and 9 o’clock positions of the pipeline cross-section, while two distributed optical fibers were placed at the 12 o’clock position, seeing Fig 15. This configuration formed two optical fiber loops: 3 o’clock + 12 o’clock and 9 o’clock + 12 o’clock.

Soil parameter monitoring: collecting field soil samples and conducting physical-mechanical tests to determine soil mechanical parameters including soil type, unit weight, cohesion, internal friction angle, elastic modulus, Poisson’s ratio, etc.

### 4.2 Experiment results

Soil samples near the sinking and lowering-in area were tested according to static simple shear tests to determine the physical index and mechanical parameter (seeing Table 1), which were provided as basic input parameters for the analytical and numerical models.

**Table 1:** The physical index and mechanical parameter of soil samples.

Sample	Soil category	Natural density *ρ*(g/cm^3^)	Specific gravity	Plasticity index	Modulus of elasticity *E*(MPa)	Cohesion *c*(kPa)	Angle of internal friction *φ*(°)	Poisson’s ratio *μ*
Sample 1	Silty clay	2.02	2.08	13.02	20.52	21.71	26.39	0.39
Sample 2	Silty clay	2.08	1.96	14.33	19.84	24.73	25.47	0.31

**Fig 16:**
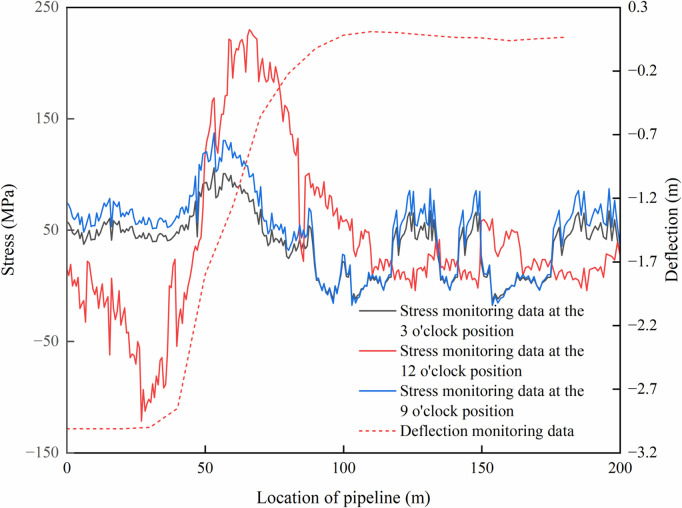
Monitoring data of axial stress and deflection when pipeline just reaching to trench bottom.

**Fig 17:**
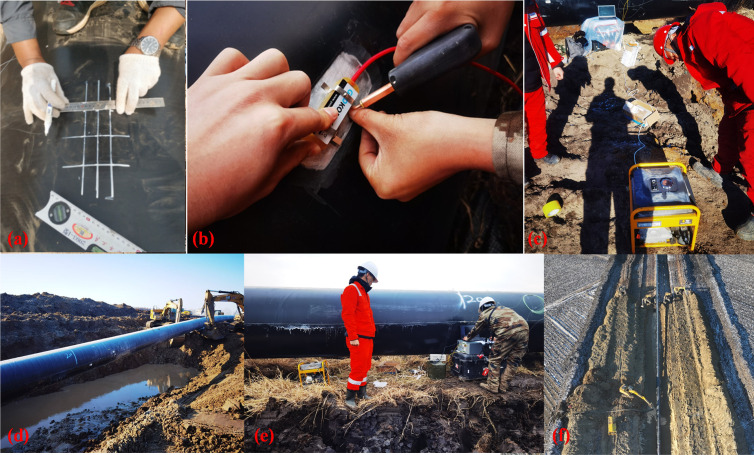
Photographs related to the on-site experiment.

Through the on-site experiment, the stress and deflection curve were obtained when the pipeline just touching to the trench bottom, as shown in Fig 16. Experimental photographs taken during the on-site experiment were presented in Fig 17.

Based on the fiber optic monitoring data, when pipeline just touched to the trench bottom, the soil compressed displacement measured at the trench excavation end has a value of Δ = 0.25m, with the arched segment length of *L*_1_ = 44.25m and suspended segment length of *L*_2_ = 61.79m, all approximating analytical results. The maximum stress of the pipeline occurred at 66 m with a value of 229.80 MPa. In addition, near the trench excavation boundary of *L*_2_ = 61.79m, the noticeable stress concentration was observed, which is consistent to numerical simulation results. Influenced by construction factors (such as pipeline horizontal offset and rotation during sinking and lowering-in), slight stress fluctuations were detected at both sides of the pipeline (3 o’clock and 9 o’clock positions) during the sinking and lowering-in, exhibiting a symmetrical distribution pattern about the zero-stress line.

## 5 Analytical model validation

### 5.1 Sinking and lowering-in

#### (1) Basic parameters.

Soil mechanical properties: unit weight of soil 20.2 kN/m³, internal friction angle 25.93°, cohesion 23.22 kPa, elastic modulus 20.18 MPa, Poisson’s ratio 0.35, yield displacement 0.00508 m, which were resulting from section 4.2.

Pipeline mechanical properties: density 7.85 × 10³ kg/m³, elastic modulus 2.06 × 10^5^ MPa, shear modulus 7.9 × 10^4^ MPa, Poisson’s ratio 0.3, yield strength 485 MPa, tensile strength 555 MPa.

Geometric parameters: soil dimensions 200 m (length) × 7 m (width) × 6 m (height), trench excavation length 100 m, trench excavation depth 2.0 m, pipeline length 200 m, pipeline outer diameter 1016 mm, wall thickness 26.2 mm.

Importantly, pipeline and soil parameters were totally identical to the analytical model, the finite element model and the on-site experiment.

#### (2) Loads and constraints.

Element and constitutive models: C3D8R eight-node linear hexahedral elements for pipeline and soil; finite sliding surface-to-surface contact between the pipeline and soil; the bilinear stress-strain hardening relationship to simulate the elastoplastic behavior of the pipeline; Mohr-Coulomb ideal elastoplastic criterion to model soil plasticity.

Boundary conditions: normal displacement constraints in the bottom and four lateral surfaces (excluding the top surface) of the soil domain; full displacement constraints at the pipeline’s right end while the left end remained free; soil-pipeline interaction through contact constraints.

Loading conditions: self-weight of the pipeline and soil; the pipeline lowering process simulated through static analysis as following: using “ model change” technique in Abaqus to kill a part of soil elements to achieve trench excavation and to model pipeline initial displacement *h*.

**Fig 18:**
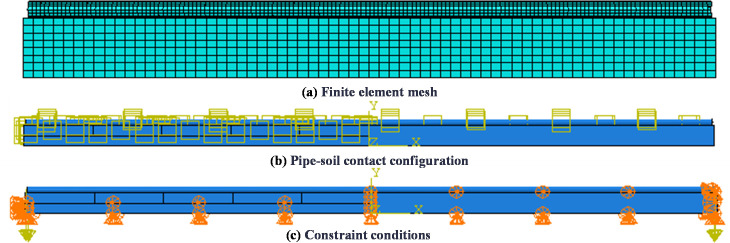
Finite element model of stress analysis during pipeline lowering-in.

Based on the above conditions, the finite element model of stress analysis for pipeline sinking and lowering-in was established by ABAQUS, as illustrated in Fig 18.

**Fig 19:**
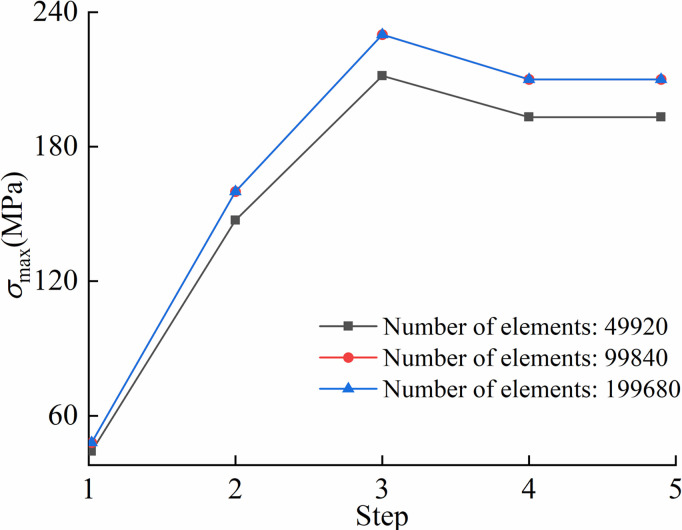
Validation of number of grids on calculation accuracy of numerical model.

The numerical model was discretized into 99,840 elements. To ensure grid independence of numerical results, a mesh sensitivity analysis was conducted. Fig 19 illustrated the variation of pipeline maximum stress under different elements.

From Fig 19, the finite element models with 99,840 and 199,680 elements demonstrated an overall agreement on pipeline maximum stress, indicating the element model provided sufficient simulation accuracy when at least covering 99,840 elements. The 49,920-element model has a certain deviation in pipeline maximum stress, compared to the other two models.

#### (3) Simulation results.

**Fig 20:**
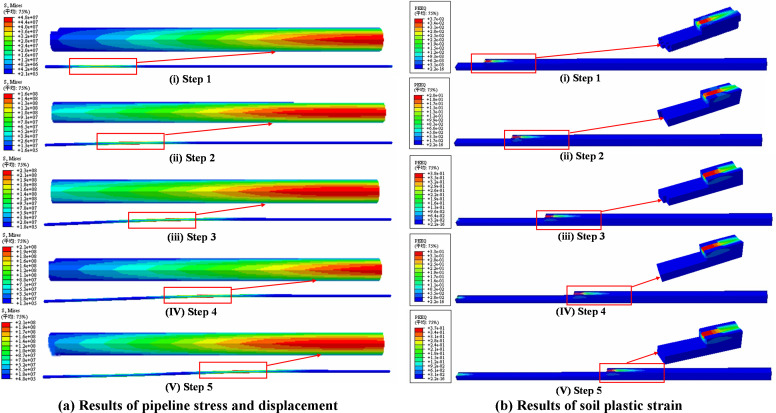
Results of pipeline stress and displacement and soil plastic strain during lowering-in. (a) Results of pipeline stress and displacement. (b) Results of soil plastic strain.

The simulation was divided into 5 displacement sub-steps to accomplish trench excavation. Fig 20 presented the pipeline stress and soil plastic strain distributions at different sub-steps.

As illustrated in Fig 20, with the progressive loading of displacement sub-steps, the pipeline stress and soil plastic strain demonstrated an initial increase followed by a slight reduction, while the displacement continued to increase. The results indicated that when the trench excavation length reached 59.6m (i.e., the third sub-step), the pipeline began to touch to the trench bottom. At this stage, the pipeline peak stress attained the maximum of 230.14 MPa, accompanied by the maximum soil plastic strain of 0.38. After touching to trench bottom, the pipeline peak stress initiated a downward trend. When finalizing 100 m of excavation length, the pipeline peak stress reduced to 211.22 MPa with corresponding soil plastic strain of 0.37, representing respective decreases of 8.22% and 2.63% compared to the maximum observed at 59.6 m of excavation length.

#### (4) Model verification.

Considering that the proposed model and code-specified model [28] were based on the condition of pipeline toughing to the trench bottom, finite element results and on-site experiment results at the moment of pipeline just reaching to the trench bottom were extracted to verify the proposed model. Importantly, the basic parameters were totally same to the analytical model, the finite element model and the on-site experiment. Specifically, the comparison parameters were the output variables of the proposed model, including the arched segment length *L*_1_, the suspended segment length *L*_2_, the maximum stress $\sigma_{\max}$, and the bending moment $M_{\max}$at the trench excavation end.

**Fig 21:**
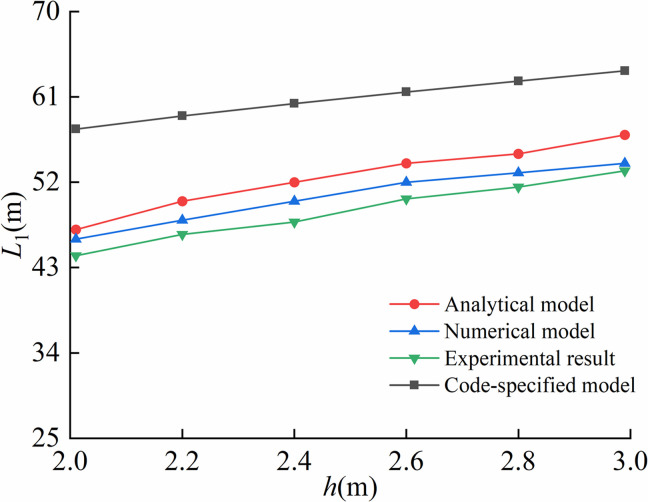
Comparison of the length of arched segment under different trench depths.

Comparison of arched segment lengths under different trench depths was illustrated in Fig 21.

**Fig 22:**
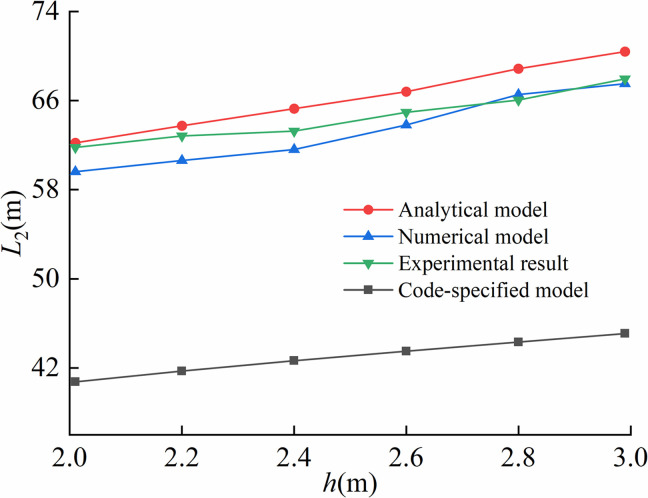
Comparison of the length of suspended segment under different trench depths.

Fig 22 presented the comparison of suspended segment lengths obtained from the proposed model, code-specified model, finite element model and on-site experiment under varying trench depths.

**Fig 23:**
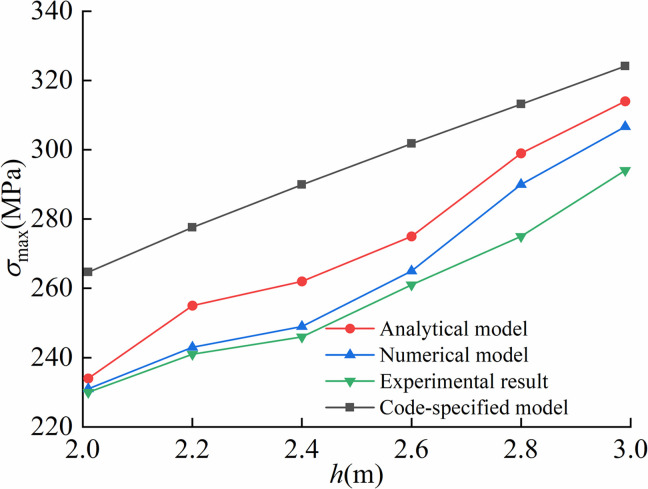
Comparison of the maximum stress under different trench depths.

Maximum stresses calculated by the proposed model, code-specified model, finite element model and on-site experiment under different trench depths were compared in Fig 23.

**Fig 24:**
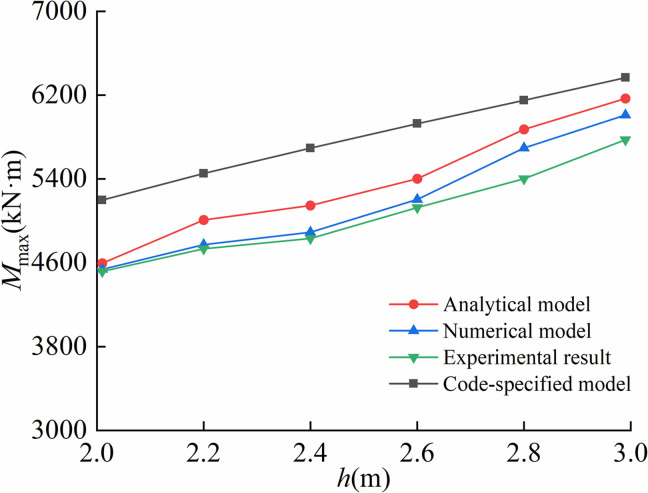
Comparison of the maximum bending moment under different trench depths.

Given that the maximum bending moment occurred at the trench excavation end, Fig 24 demonstrated the comparative analysis of maximum bending moments predicted by the proposed model, code-specified model, finite element model and on-site experiment across different trench depths.

As illustrated in Figs 21–24, the proposed analytical model showed the better agreement to finite element model than code-specified model. This is mainly because the soil displacement Δ at the trench excavation end in the proposed model better simulated the actual soil compression during pipeline lowering-in. In contrast, the existing code-specified model imposed the vertical constraint directly at the trench excavation end, failing to account for realistic soil compression behavior, thereby leading to reduced computational precision. Results indicated that the soil displacements at the end of excavation of pipeline trenches were respectively 0.21 m, 0.22 m, 0.25 m for the proposed model, the finite element model and the on-site experiment, while equaling to 0 m for the code-specified model.

For the arched segment length *L*_1_, the suspended segment length *L*_2_, the maximum stress $\sigma_{\max}$, and the bending moment $M_{\max}$, the maximum relative errors of the proposed analytical model compared to finite element model were 5.56%, 5.96%, 5.35%, 7.36%, and the ones of the proposed analytical model with on-site experiment were 8.79%, 4.27%, 8.68%, 8.72%, respectively, far lower than the one of 51.67% led by the code-specified model. Overall, the proposed model achieved superior computational accuracy over existing code-specified model, demonstrating enhanced applicability in engineering practices.

### 5.2 Lifting and lowering-in

#### (1) Basic parameters.

The geometry parameters in finite element model were as follows: the soil dimensions of 150 m × 7.2 m × 6.6 m; the trench of length of 120 m and depth of 2.6 m, located at the center of the soil domain; the pipeline length of 150 m; the outer diameter of the pipeline of 1016 mm; the wall thickness of the pipeline of 21 mm; span of lifting slings of 15 m, totaling 8 lifting slings, with the first sling positioned at the trench end. The mechanical parameters for each component material were listed in Table 2.

**Table 2:** Mechanical parameters used in the finite element model.

Material type	Elastic modulus (MPa)	Poisson’s ratio	Density (kg/m^3^)	Yield strength (MPa)	Tensile strength (MPa)	Internal friction angle (°)	Dilation angle (°)	Cohesion (kPa)
Soil	60	0.25	1.78 × 10^3^	24	/	25	4	12
Pipe	210 × 10^6^	0.3	7.85 × 10^3^	485MPa	555MPa	/	/	/
Sling	210 × 10^6^	0.3	7.85 × 10^3^	485MPa	555MPa	/	/	/

#### (2) Loads and constraints.

Element and constitutive relation: C3D8R 8-node linear hexahedral elements for the pipeline, soil, and lifting slings; the bilinear stress-strain hardening relationship to simulate the elastoplastic behavior of the pipeline, while the ideal elastoplastic Mohr-Coulomb criterion to characterize the plastic deformation of the soil.

Boundary conditions: normal displacement constraints in all lateral surfaces of the soil except for the top surface and trench bottom; full displacement constraints at the left end of the pipeline, while free at the right end being; pre-defined finite-sliding contact surfaces between the pipeline and lifting slings; pre-defined finite-sliding contact surfaces between the pipeline and soil.

Loading conditions: gravitational loads for the pipeline, soil, and lifting slings; lifting in the vertical plane by applying *y*-direction displacements to the slings, followed by horizontal offset through *x*-direction displacements;

Especially, the completed pipeline lifting and lowering-in was realized through sequential application of specified *y*- and *x*-direction displacements as detailed in Table 3.

**Table 3:** Lifting point displacement.

Displacements	Lifting point 1	Lifting point 2	Lifting point 3	Lifting point 4	Lifting point 5	Lifting point 6	Lifting point 7	Lifting point 8
Horizontal displacement (m)	−3.6	−3.3	−2.88	−2.39	−1.85	−1.34	−0.88	−0.51
Vertical displacement (m)	−2.6	−1.76	−0.98	−0.29	0.19	0.41	0.41	0.25

**Fig 25:**
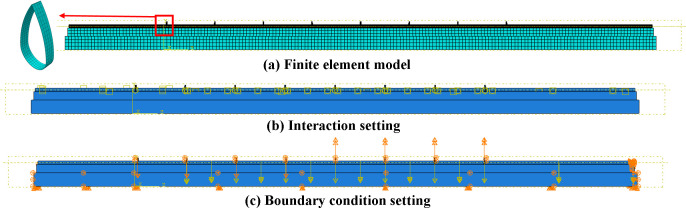
Finite element model of lifting and lowering-in for pipeline. (a) Finite element model. (b) Interaction setting. (c) Boundary condition setting.

Based on the above parameters and conditions, the finite element model for stress analysis during pipeline lowering-in was established by ABAQUS. Detailed mesh configurations, contact definitions, and boundary conditions were illustrated in Fig 25.

#### (3) Simulation results.

**Fig 26:**
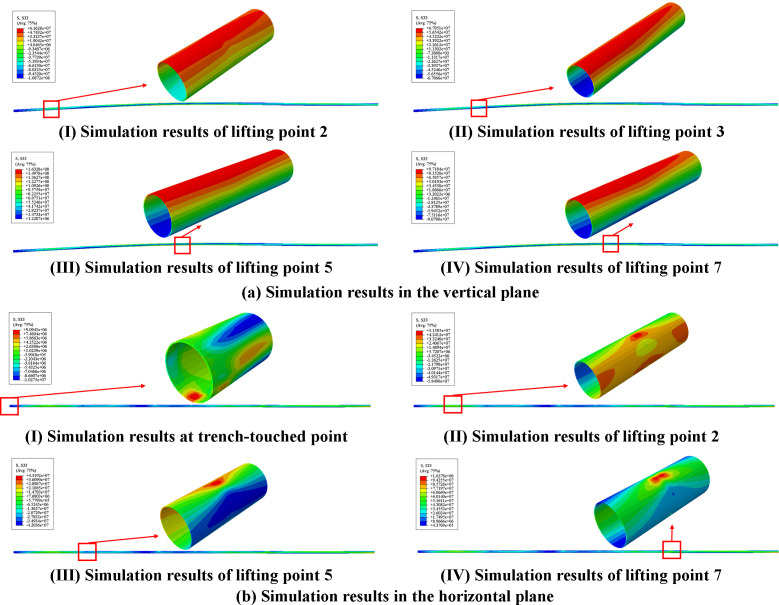
Simulation results of the lifting and lowering-in for pipeline. (a) Simulation results in the vertical plane (I) Simulation results of lifting point 2. (II) Simulation results of lifting point 3. (III) Simulation results of lifting point 5 (IV) Simulation results of lifting point 7. (b) Simulation results in the horizontal plane (I) Simulation results at trench-touched point (II) Simulation results of lifting point 2 (III) Simulation results of lifting point 3 (IV) Simulation results of lifting point 7.

The simulation comprised two displacement loading sub-steps, to achieve the completed pipeline lowering-in through prescribed vertical and horizontal displacements. Numerical results at critical contact points and selected lifting positions were presented in Fig 26.

**Fig 27:**
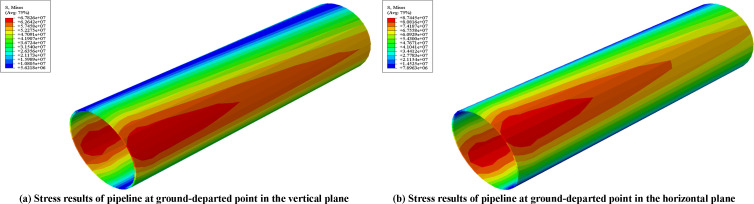
Simulation results of pipeline stress at the ground-departed point. (a) Stress results of pipeline at ground-departed (b) Stress results of pipeline at ground-departed point in the vertical plane point in the horizontal plane.

Numerical results indicated that the pipeline stress and contact stress at pipeline-sling interfaces remained relatively low. During lifting in the vertical plane, the pipeline stress increased significantly after applying specified vertical displacements in Table 3. The maximum stress of 163.28 MPa occurred at the upper position of pipeline cross-section at the 5th lifting point. Meanwhile, the pipeline stress at ground-departed position reached 67.83 MPa (seeing Fig 27(a)). In the horizontal plane, the pipeline peak stress of 102.78 MPa emerged at the upper position of pipeline cross-section at the 8th lifting point, with pipeline stress at ground-departed point maintaining approximately 87.44 MPa (seeing Fig 27(b)).

#### (4) Model verification.

The proposed analytical model enabled calculation of lifting forces and pipeline stresses in a vertical and horizontal plane. In view of this, lifting forces and pipeline stresses from finite element analysis during corresponding loading steps were extracted. Notably, lifting forces were derived through integration of contact stresses over element areas at sling-pipeline interfaces.

**Fig 28:**
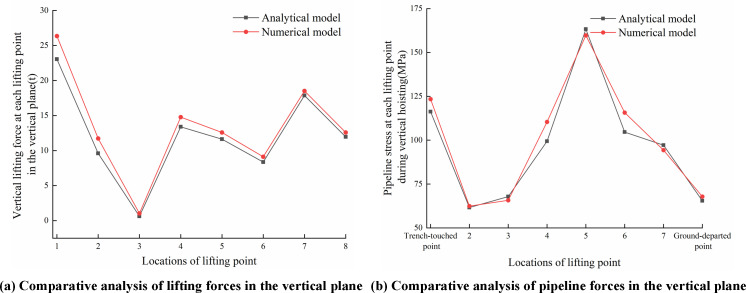
Comparisons of lifting point force and pipeline stress in the vertical plane. (a) Comparative analysis of lifting forces in the (b) Comparative analysis of pipeline stress in the vertical plane vertical plane.

**Fig 29:**
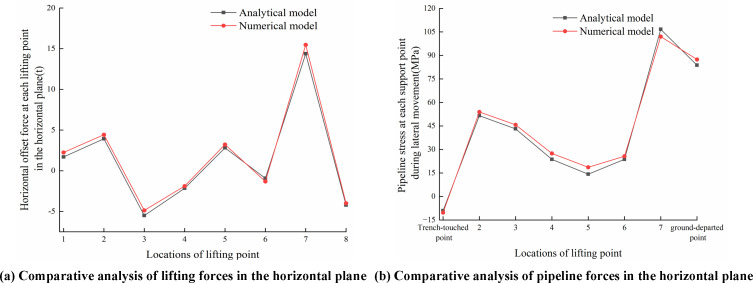
Comparisons of lifting point force and pipeline stress in the horizontal plane. (a) Comparative analysis of lifting forces in the (b) Comparative analysis of pipeline stress in the horizontal plane horizontal plane.

**Fig 30:**
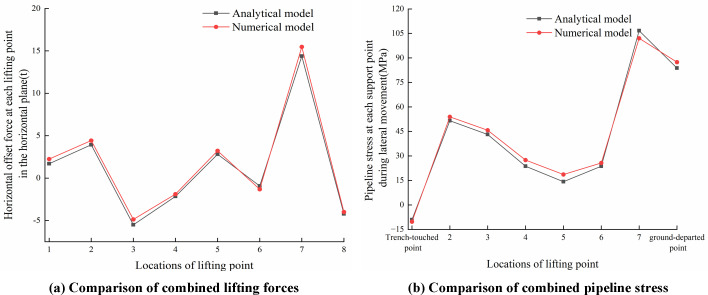
Comparisons of combined lifting force and combined stress. (a) Comparison of combined lifting forces (b) Comparison of combined stress.

Figs 28–30 compared analytical and numerical results for lifting forces and pipeline stresses at each lifting point in the vertical and horizontal planes, as well as combined lifting forces and combined pipeline stresses.

As seen from Figs 28–30, the theoretical results calculated by the analytical model demonstrated conservative characteristics compared to numerical results, while maintaining generally good agreement with finite element results. The primary discrepancy between analytical and numerical solutions originated from the simplified assumption in the analytical model, i.e., simplifying sling constraints as concentrated forces to lead to difference from the actual surface-to-surface contact between slings and pipelines. Selecting vertical lifting forces in the vertical plane, the horizontal offset forces in the horizontal plane, combined lifting forces, and combined stresses at each lifting point as comparison parameters, the maximum relative errors between the analytical model and finite element model were 7.63%, 8.59%, 3.74%, 6.44%, 9.51% and 8.13% respectively. Specifically, comparative results of pipeline stresses at trench-touched point and ground-departed point between analytical and numerical solutions were detailed in Table 4.

**Table 4:** Comparisons of pipeline stress at the trench-touched point and the ground-departed point.

Location	Analytical solution			Numerical solution		
	Stress in vertical plane/ MPa	Stress in horizontal plane/ MPa	Combined stress/ MPa	Lifting in vertical plane/ MPa	Stress in horizontal plane/ MPa	Combined stress/ MPa
Trench-touched point	116.26	−9.69	116.25	123.4	−10.28	124.8
Ground-departed point	65.42	83.76	106.28	67.83	87.44	110.66

The results indicated that relative errors between the proposed model and numerical model for pipeline stresses at trench-touched point and ground-departed point were as follows: 8.59% and 3.68% (in the vertical plane), 5.73% and 4.39% (in the horizontal plane), 6.85% and 4.12% (combined stresses) respectively.

## 6 Conclusions

(1) Considering the soil compressive displacement at the end boundary of being-dug trench during pipeline sinking and lowering-in, the analytical model for stress analysis for pipeline lowering-in was established. Meanwhile, it is considered for the extreme displacement scenarios at first and last lifting points to determine the control condition for calculation of lifting force and pipeline stress, and then pipeline bending moment at lifting point and then lifting force were obtained to establish the analytical model of stress analysis for pipeline lifting and lowering-in.
(2) By the finite element model, the analytical model for sinking and lowering-in was validated. Taking the length of arched segment, the length of suspended segment, the maximum stress and the bending moment as comparison variables, the maximum errors were 5.56%, 5.96%, 5.35%, 7.36% between the sinking and lowering-in model and the finite element model, while were 8.79%, 4.27%, 8.68%, 8.72% between the sinking and lowering-in model and the on-site experiment.
(3) The maximum relative errors between the lifting and lowering-in model and finite element model were 7.63%, 8.59%, 3.74%, 6.44%, 9.51% and 8.13%, respectively, considering the lifting force and pipeline stress in the vertical plane, the horizontal offset force and pipeline stress in the horizontal plane, and the combined lifting force and combined stress as comparison parameters, and meanwhile the analytical results showed the overall agreement to numerical solutions at the trench-touched point and the ground-departed point, with the relative errors of 8.59% and 3.68% (in the vertical plane), 5.73% and 4.39% (in the horizontal plane), 6.85% and 4.12% (combined stress), respectively.

## Limitations

There is only the on-site experiment for sinking and lowering-in to be carried out, to validate the corresponding analytical model. In contrast, there are no the on-site experiments to be performed for lifting and lowering-in, which, to some extent, made the validations on the other two analytical models to be minor deficiency.

### Permit exemption statement

“No specific permits were required for the analytical model of stress analysis for pipeline lowering-in during construction because:

(1) The research exclusively involved theoretical calculations and numerical simulations based on publicly available engineering parameters, with no field sampling or interaction with protected species;
(2) The study utilized open-source data from standardized pipeline specifications (e.g., ASME B31J-2017 stress intensification factors) without accessing restricted databases.

## Supporting information

S1 Fig Stress testing equipments for on-site experiment of pipeline sinking and lowering-in. (a) Vibrating wire strain sensor for monitoring the maximum stress in pipelines (b) Distributed optical fibers for monitoring the stress distribution in pipelines (c) M350 RTK photographic drone for monitoring pipeline deflection curves (d) Remote controller for M350 RTK photographic drone. (TIF)

S2 Fig Soil parameter testing. (a) Soil sample (b) Static direct shear test to obtain soil shear strength.
(TIF)

S3 Fig On-site experiment progress. (a) Local deformation of pipelines with enlarged scale. (b) Top view of overall deformation of pipelines.
(TIF)

S1 Data Excel spreadsheet of raw data used to generate Fig 28 (a)–(b). (XLSX)

S2 Data Excel spreadsheet of raw data used to generate Fig 29 (a)–(b). (XLSX)

S3 Data Excel spreadsheet of raw data used to generate Fig 30 (a)–(b). (XLSX)

## References

[1] Zhang LS, Zhao XB, Yan XZ, Yang XJ. Elastoplastic analysis of mechanical response of buried pipelines under strike-slip faults. Int J Geomech. 2017;17(4):04016109-1–04016109-13.

[2] Zhang LS, Fang ML, Pang XF, Yan XZ, Cao YG. Mechanical behavior of pipelines subjecting to horizontal landslides using a new finite element model with equivalent boundary springs. Thin Wall Struct. 2018;124:501–13.

[3] Cheng HL, Long SH, Wang NT, Cao XD. Technique of pipeline-settled ditching in small caliber long distance pipeline construction at high groundwater level. J Beijing Inst Petro-Chem Technol. 2014;22(1):8–11. (In Chinese)

[4] Kang WP, Wang Y, Li ZH. Application of pipe sinking technique in valley pipeline construction. Oil Gas Storage Transport. 2010;29(7):550–2. (In Chinese)

[5] Yu JJ. Optimal selection of lifting points of AP1000 long pipe modules. Constr Des Proj. 2013;(11):112–4. (In Chinese)

[6] Zhang L, Li DD, Peng SB. Application of lowering pipeline at single side to long-distance pipeline construction in desert areas. Nat Gas Oil. 2011;29(4):23–5. (In Chinese)

[7] Zhang LS, Zhao XB, Yan XZ, Yang XJ. A new finite element model of buried steel pipelines crossing strike-slip considering equivalent boundary springs. Eng Struct. 2016;123:30–44.

[8] Lan XB, Yang YB, Fang ML, Zhao LX, Zhu HR. Mechanical analysis on immersed ditching pipeline. J Safety Sci Technol. 2023;19(6):112–8. (In Chinese)

[9] Shi T, Wang YB, Jin GY, Liu XB, Zhang H, Huang QY. Analysis of stress response trends and critical depth of large diameter X80 pipe lowering-in. Petrol Tubular Goods Instrum. 2023;9(5):32–40. (In Chinese)

[10] Meng J, Kang K. Immersed ditch technical countermeasures research of pipeline through water network area. Pipeline Technique Eq. 2017;24(3):1–3.

[11] Wang FH, Lian ZH, Lin TJ. Theoretical analysis and numerical simulation of pipeline stress with lowering pipeline on both sides in water network areas. China Petro Mach. 2015;43(7):116–120. (In Chinese)

[12] Gu QY, Liu CJ, Lin ZM. Stress analysis of sinking pipeline into trench for long distance pipeline. Petro Eng Constr. 2019;45(2):16–19. (In Chinese)

[13] Liu YQ, Wu YL, Zhang ZY. Feasibility of lowering-in of horizontal continuous cold bending pipes in China-Russia Eastern Gas Pipeline. Oil Gas Storage Transport. 2020;39(2):215–221. (In Chinese)

[14] Liu YQ, Yu ZF, Qi WP. Numerical simulation of sinking and lowering of China-Russia eastern gas pipeline. China Petro Mach. 2019;47(3):118–23. (In Chinese)

[15] Duan DM, Jurca T, Zhou C. A stress check procedure for pipe lowering-in process during pipeline construction. In: Proceedings of the IPC, 10th International Pipeline Conference; 2014 Sep 29–Oct 3; Calgary, AB, Canada. 2014.

[16] Hwang W, Lee JS. Analytical model for the structural behavior of pipelines during lowering-in. Appl Sci. 2019;9:2595.

[17] Wang JW, Wang YY, Bruce WA, Rapp S, Scoles R. Development of lifting and lowering-in plan for the control of construction stresses. Proceedings of the 2020 13th International Pipeline Conference; 2020 Sep 28–30; Virtual, Online. 2020.

[18] Sen M, Zhou J. Evaluation of pipeline stresses during line lowering. In: Proceedings of the 7th International Pipeline Conference (IPC 2008); 2008 Sep 29–Oct 3; Calgary, AB, Canada. 2008.

[19] Carneiro D, Rosito R, Carvalho G. Increasing productivity and safety of pipe lowering through finite element analyses. 8th International Pipeline Conference (IPC 2010); 2010 Sep 27–Oct1; Calgary, AB, Canada. 2010.

[20] Zhang F, Liu M, Wang YY. Stress analysis of lifting and lowering-in process. Proceedings of The 10th International Pipeline Conference; 2014 Sep 29–Oct 3; Calgary, AB, Canada. 2014.

[21] Liu M, Wang YY, Rogers G. Stress analysis of pipe lowering-in process during construction. ASME International Pipelines Conference; 2008 Sep 29–Oct 3; Calgary, AB, Canada. 2008.

[22] Scott C, Etheridge B, Vieth P. An analysis of the stresses incurred in pipe during laying operations. In: Proceedings of the 7th International Pipeline Conference (IPC 2008); 2008 Sep 29–Oct 3; Calgary, AB, Canada. 2008.

[23] Alexander C, Scrivner R. Analysis of girth welds in pipelines subjected to lifting loads. Proceedings of IPC 2006 6th International Pipeline Conference; 2006 Sep 25-29; Calgary, Alberta, Canada. 2006.

[24] Trapper PA. A numerical model for geometrically nonlinear analysis of a pipe-lay on a rough seafloor. Ocean Eng. 2022;252:111146.

[25] Xu P, Gong SF. Pipelay parametric investigation of pipeline dynamic behaviors for deepwater S-lay operation. Ships Offshore Struc. 2020;15(10):1141–55.

[26] Lenci S, Callegari M. Simple analytical models for the J-lay problem. Acta Mech. 2005;178:23–39.

[27] Xu P, Du Z, Huang F, Javanmardi A. Numerical simulation of deepwater S-lay and J-lay pipeline using vector form intrinsic finite element method. Ocean Engineering. 2021;234:109039. doi: 10.1016/j.oceaneng.2021.109039

[28] China Petroleum Pipeline Engineering Company. Specification for oil and gas transportation pipeline sinking and lowering in Q/SY CDJ 0387-2014. Beijing; 2015.

[29] Hetenyi M. Beams on Elastic Foundation. University of Michigan Press; 1946.

[30] Kerr AD. Elastic and viscoelastic foundation models. J Appl Mech. 1964;31:491–8.

[31] Zhang L, Xie Y, Yan X, Yang X. An elastoplastic semi-analytical method to analyze the plastic mechanical behavior of buried pipelines under landslides considering operating loads. Journal of Natural Gas Science and Engineering. 2016;28:121–31. doi: 10.1016/j.jngse.2015.11.040

[32] Zhang LS, Zhao XB, Yan XZ, Yang XJ. A semi-analytical method of stress-strain analysis of buried steel pipelines under submarine landslides. Appl Ocean Res. 2016;59:38–52.

[33] The American Society of Civil Engineers. Guidelines for the seismic design of oil and gas pipeline systems. New York; 1984.

