# Supplementary figures and images for: Analytical model of stress analysis for pipeline lowering-in during construction
Source: PLoS One. 2025 Jul 1;20(7):e0325123. doi: 10.1371/journal.pone.0325123 (PMC12212509; doi:10.1371/journal.pone.0325123)

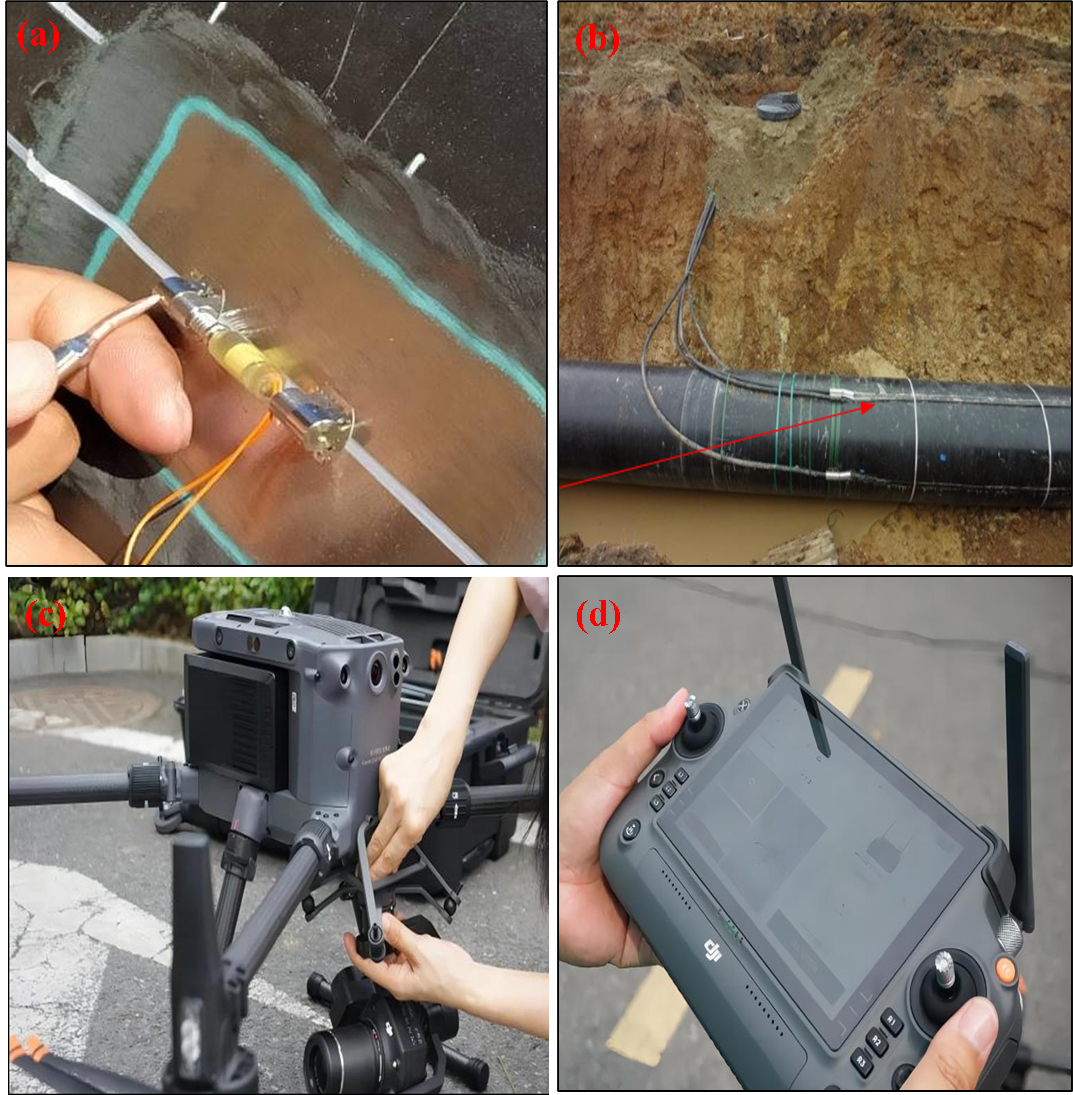

Supplement: S1 Fig — (TIF) [file pone.0325123.s001.tif]

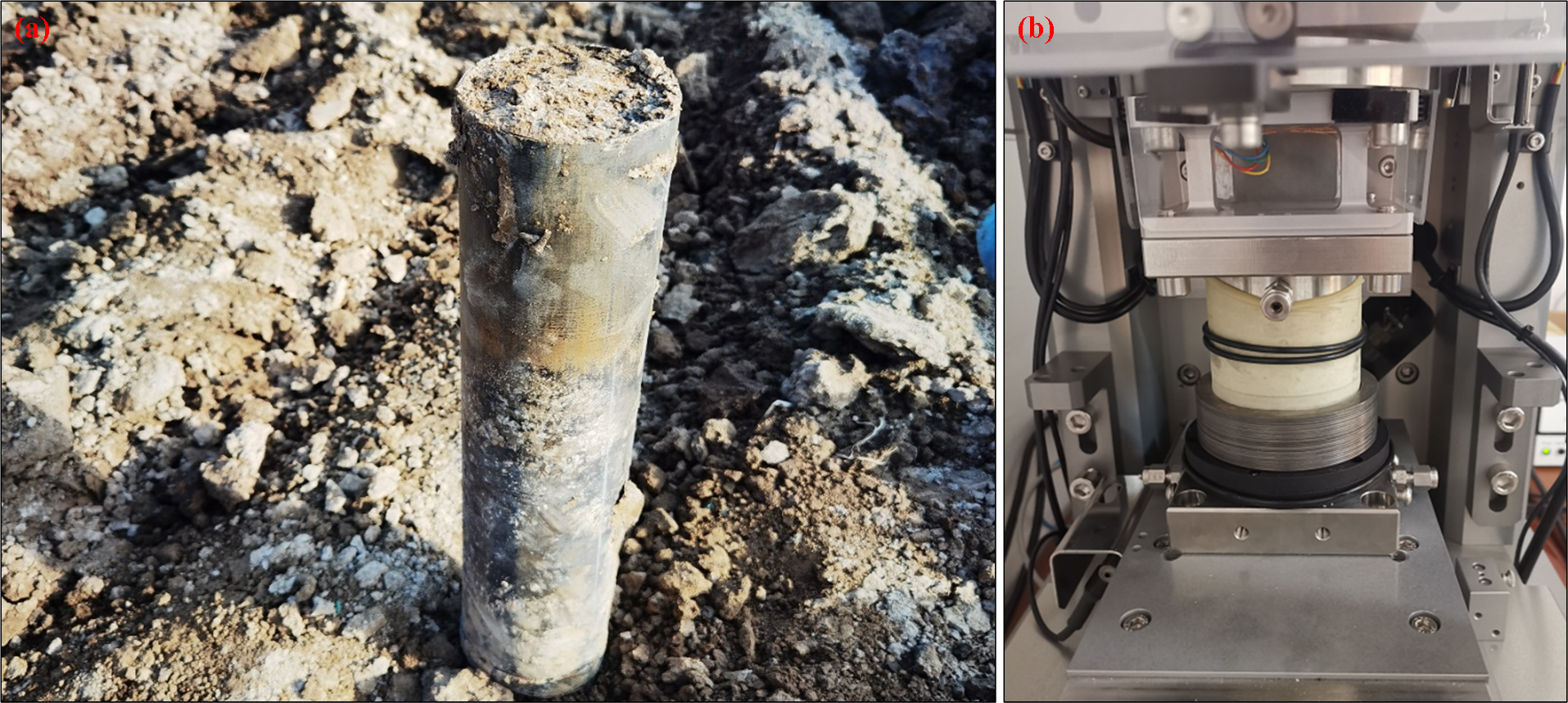

Supplement: S2 Fig — (a) Soil sample (b) Static direct shear test to obtain soil shear strength. (TIF) [file pone.0325123.s002.tif]

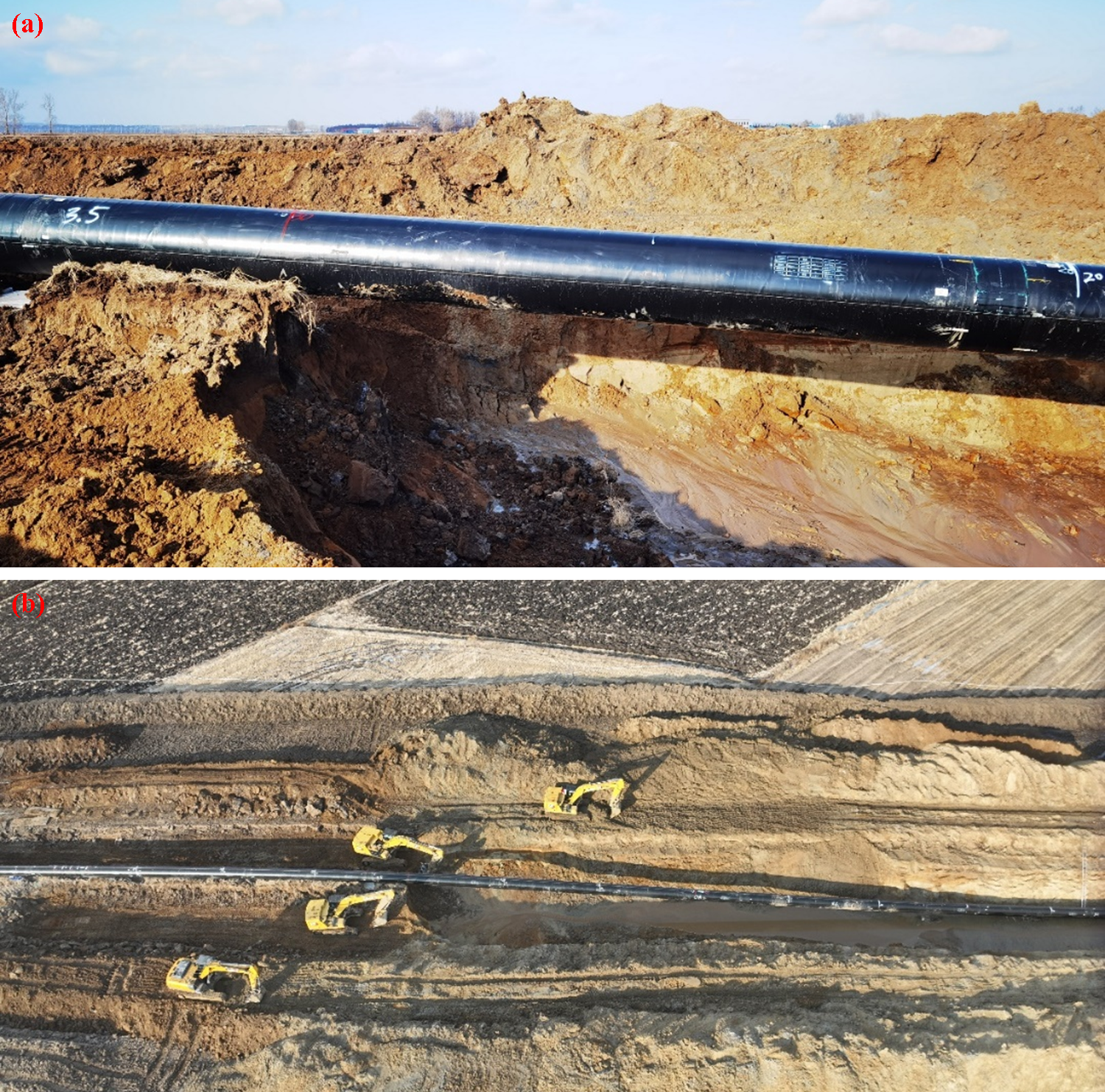

Supplement: S3 Fig — (a) Local deformation of pipelines with enlarged scale. (b) Top view of overall deformation of pipelines. (TIF) [file pone.0325123.s003.tif]
